# Developmentally-Regulated Excision of the SPβ Prophage Reconstitutes a Gene Required for Spore Envelope Maturation in *Bacillus subtilis*

**DOI:** 10.1371/journal.pgen.1004636

**Published:** 2014-10-09

**Authors:** Kimihiro Abe, Yuta Kawano, Keito Iwamoto, Kenji Arai, Yuki Maruyama, Patrick Eichenberger, Tsutomu Sato

**Affiliations:** 1 Research Center of Micro-Nano Technology, Hosei University, Koganei, Tokyo, Japan; 2 Department of Frontier Bioscience, Hosei University, Koganei, Tokyo, Japan; 3 Center for Genomics and Systems Biology, New York University, New York, New York, United States of America; University of Geneva Medical School, Switzerland

## Abstract

Temperate phages infect bacteria by injecting their DNA into bacterial cells, where it becomes incorporated into the host genome as a prophage. In the genome of *Bacillus subtilis* 168, an active prophage, SPβ, is inserted into a polysaccharide synthesis gene, *spsM*. Here, we show that a rearrangement occurs during sporulation to reconstitute a functional composite *spsM* gene by precise excision of SPβ from the chromosome. SPβ excision requires a putative site-specific recombinase, SprA, and an accessory protein, SprB. A minimized SPβ, where all the SPβ genes were deleted, except *sprA* and *sprB*, retained the SPβ excision activity during sporulation, demonstrating that *sprA* and *sprB* are necessary and sufficient for the excision. While expression of *sprA* was observed during vegetative growth, *sprB* was induced during sporulation and upon mitomycin C treatment, which triggers the phage lytic cycle. We also demonstrated that overexpression of *sprB* (but not of *sprA*) resulted in SPβ prophage excision without triggering the lytic cycle. These results suggest that *sprB* is the factor that controls the timing of phage excision. Furthermore, we provide evidence that *spsM* is essential for the addition of polysaccharides to the spore envelope. The presence of polysaccharides on the spore surface renders the spore hydrophilic in water. This property may be beneficial in allowing spores to disperse in natural environments via water flow. A similar rearrangement occurs in *Bacillus amyloliquefaciens* FZB42, where a SPβ-like element is excised during sporulation to reconstitute a polysaccharide synthesis gene, suggesting that this type of gene rearrangement is common in spore-forming bacteria because it can be spread by phage infection.

## Author Summary

Integration of prophages into protein-coding sequences of the host chromosome generally results in loss of function of the interrupted gene. In the endospore-forming organism *Bacillus subtilis* strain 168, the SPβ prophage is inserted into a previously-uncharacterized spore polysaccharide synthesis gene, *spsM*. In vegetative cells, the lytic cycle is induced in response to DNA damage. In the process, SPβ is excised from the genome to form phage particles. Here, we demonstrate that SPβ excision is also a developmentally-regulated event that occurs systematically during sporulation to reconstitute a functional *spsM* gene. Following asymmetric division of the sporulating cell, two cellular compartments are generated, the forespore, which will mature into a spore, and the mother cell, which is essential to the process of spore maturation. Because phage excision is limited to the mother cell genome, and does not occur in the forespore genome, SPβ is an integral part of the spore genome. Thus, after the spores germinate, the vegetative cells resume growth and the SPβ prophage is propagated vertically to the progeny along with the rest of the host genome. Our results suggest that the two pathways of SPβ excision support both the phage life cycle and normal sporulation of the host cells.

## Introduction

Genetic information is normally preserved across generations in living organisms. However, genomic integrity is sometimes dramatically challenged by DNA rearrangement events, such as homologous recombination, viral genome integration, and transposon spreading. These DNA rearrangements contribute to genetic diversification in the evolutionary history of life on Earth. Importantly, some of these rearrangements are programmed to occur at specific sites and times during cellular differentiation and play crucial developmental roles in a variety of organisms. The best-known example is the rearrangement of immunoglobulin genes in the B lymphocytes of the vertebrate immune system. The assembly in different combinations of the variable (V), diversity (D), and joining (J) exons of the immunoglobulin gene generates antigen receptors with extremely diverse binding specificities [1].

DNA rearrangements also modulate gene expression in bacteria during cellular differentiation. For example, during differentiation to a heterocyst, which is a cell type that fixes atmospheric nitrogen, bacteria of the *Anabaena* genus have the ability to reconstitute the disrupted *nifD*, *fdxN*, and *hupL* genes that are normally inactive in photosynthetic cells [2]–[5]. In the sporulating Gram-positive bacterium *Bacillus subtilis*, the *sigK* gene, which encodes the sporulation sigma factor σ^K^, is interrupted by the phage-like element *skin*. During sporulation, *skin* is excised and a functional composite *sigK* gene is produced [6].

*B. subtilis* cells produce endospores in response to nutrient starvation. The *B. subtilis* spore envelope is characterized by a succession of concentric layers of chemically distinct composition: the cortex is a peptidoglycan layer assembled between the inner and outer spore membranes, while the coat is an external proteinaceous layer, which can be further subdivided into an inner coat layer and an outer coat layer [7], [8]. An additional layer called the spore crust was recently discovered outside the outer coat [8]–[10]. Spore formation in *B. subtilis* has been studied extensively as a model system for cellular differentiation. The process begins with an asymmetric division of the sporulating cell, thus producing two compartments of unequal size, each containing a copy of the genome. The two compartments will differentiate into specific cell types: the forespore and the larger mother cell. During sporulation, a cascade of sporulation sigma factors governs gene expression in a temporally controlled, cell-specific manner [11]–[13]. During the early stages of sporulation, gene expression is controlled by σ^F^ in the forespore and σ^E^ in the mother cell, whereas σ^G^ (in the forespore) and σ^K^ (in the mother cell) control the later stages of the developmental program. The σ^K^-encoding gene, *sigK*, is disrupted by *skin* thereby splitting the gene into two protein coding sequences, *spoIVCB* (5′-end of *sigK*) and *spoIIIC* (3′-end of *sigK*) [6]. A site-specific DNA recombinase, SpoIVCA, promotes excision of *skin* from the chromosome and the joining in frame of *spoIVCB* and *spoIIIC* to reconstitute a functional *sigK* gene [6], [14]–[16]. The *spoIVCA* gene is located in the *skin* element and is expressed exclusively in the mother cell during sporulation under the control of σ^E^. The rearranged mother cell chromosome is not transmitted to the progeny because the mother cell undergoes autolysis at the end of sporulation to release the mature spore (whose genome has not been rearranged) in the environment. A similar rearrangement of the *sigK* gene was observed in the pathogenic spore-forming bacterium *Clostridium difficile* [17]. This type of DNA rearrangement was thought to be a unique case because no examples other than *sigK* had been reported in spore-forming bacteria. However, we recently characterized two other cases of novel intervening sequence elements in mother cell-expressed sporulation genes, *vfbin* in the *spoVFB* gene of *Bacillus weihenstephanensis* KBAB4 and *vrin* in the *spoVR* gene of *Geobacillus thermoglucosidasius* C56-YS93 [18]. These findings suggest that DNA rearrangements may be common in the mother cell genome of spore-forming species, prompting us to embark in a systematic analysis of intervening sequence elements in spore-forming bacteria.

*B. subtilis* 168 contains 10 prophage-like elements [19]. Of these 10 elements, only *skin* and SPβ are inserted into protein-coding regions. SPβ is integrated into *spsM* (spore polysaccharide synthesis protein M), thus producing two gene fragments, *yodU* (5′-end of *spsM*) and *ypqP* (3′-end of *spsM*). The *yodU* and *ypqP* genes are expressed during sporulation under the control of σ^K^ [20]–[22]. The most significant difference between *skin* and SPβ is that *skin* is a cryptic phage, whereas SPβ is an active prophage. SPβ usually stays in the dormant state (lysogenic cycle). However, when the SOS response is induced by DNA damage, specific genes in the SPβ genome are activated to generate virions that are released after lysis of the host cell (lytic cycle) [23]. A putative site-specific recombinase SprA (SPβ site-specific recombination factor A; formerly *yokA*) encoded in the SPβ prophage region is a candidate to promote SPβ excision from the host genome [23]. Nevertheless, the requirement for SprA in SPβ excision had not been investigated until now and the mechanism of excision is poorly understood. In the present study, we examined the fate of SPβ during sporulation. We showed that SPβ was excised from the mother cell genome, thus producing a composite *spsM* gene. We also investigated the biological function of SpsM and discovered significant changes in the surface properties of spores produced by mutant strains unable to reconstitute a functional *spsM* gene.

## Results

### *yodU* and *ypqP* Encode the N- and C-Terminal Portions of SpsM in *B. subtilis* 168

In the genome of *B. subtilis* 168, the SPβ prophage is located between two open reading frames (ORFs), *yodU* (NCBI gene locus tag BSU19810) and *ypqP* (BSU21670). Amino acid (aa) sequence alignment and comparison to non-SPβ lysogenic *B. subtilis* strains, such as BEST195 (NCBI reference sequence no. NC_017196), showed that YodU (140 aa) and YpqP (207 aa) corresponded to the N- and the C-terminal portions of SpsM (Figure S1A). An overlapping 5-aa sequence “TDKAV” was observed at the C-terminus of YodU and at the N-terminus of YpqP. This sequence corresponds to the translation of the nucleotide sequence of the attachment site for SPβ. When the aa sequences of YodU and YpqP were joined at the overlapping sequence, the composite SpsM aa sequence was identical to that of strain BEST195, thereby indicating that *B. subtilis* 168 *spsM* does not contain any mutations (non-sense, missense, deletions or insertions). SpsM is a 341-aa protein, which contains a Polysacc_synt_2 domain (Pfam accession number, PF02719) in the 18–296-aa region. This domain was first observed in *Staphylococcus aureus* CapD [24], and is shared among bacterial polysaccharide biosynthesis proteins, such as *Campylobacter jejuni* WlaL (putative sugar epimerase/dehydrogenase) [25] and several sugar epimerases. SpsM shared 38% identity with a *B. subtilis* paralog, EpsC [26]. EpsC is an UDP–sugar epimerase encoded by the *epsC* locus and is essential for the production of extracellular polysaccharide (EPS) during biofilm formation [26]. *B. subtilis* SpsM has not been previously characterized, but the conserved domain and similarity to EpsC suggest that SpsM is a sugar epimerase likely to be involved in polysaccharide synthesis. However, a capsular polysaccharide has yet to be identified in vegetative cells of *B. subtilis*. Considering that *yodU* (the 5′-segment of *spsM*) and *ypqP* (the 3′-segment of *spsM*) were identified as sporulation genes in recent transcriptomic analyses of *B. subtilis* 168 [21], [22], we postulated that *spsM* is involved in the synthesis of the spore polysaccharide. Similar transcriptional profiling results were obtained in the PY79 strain of *B. subtilis*, which is derived from 168, but cured of SPβ, where intact *spsM* was reported as a σ^K^-dependent gene [20]. As a whole, this information led us to hypothesize that in *B. subtilis* 168 the *spsM* rearrangement occurs during sporulation to allow production of spore polysaccharide.

### Reconstitution of *spsM* upon MMC Treatment and during Sporulation

**Figure 1:**
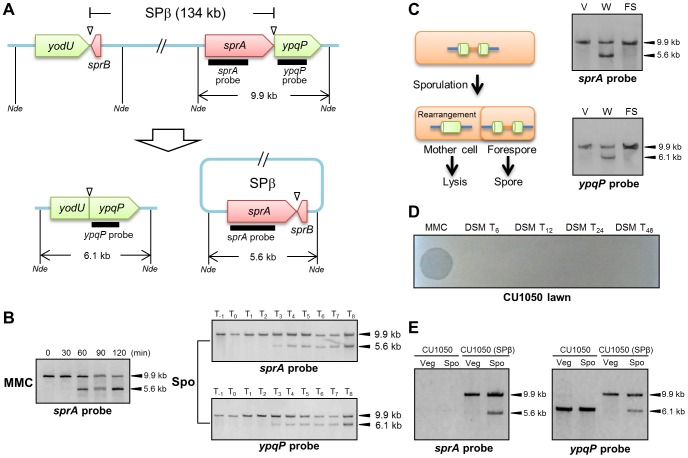
DNA rearrangement at the *spsM* locus. (A) Diagram showing SPβ excision in *Bacillus subtilis* 168. The thick lines indicate the location of the digoxigenin (DIG)-labeled probes used for Southern blotting. *Nde* indicates *Nde*I sites. Triangles point to the attachment sites for SPβ. (B) SPβ excision upon mitomycin C (MMC) treatment and during sporulation. Left panel shows induction of SPβ excision by MMC treatment. *B. subtilis* 168 cells were grown in LB medium. Vegetative cells in the early log phase (OD_600_ = 0.25) were treated with 0.5 µg/ml MMC. Time 0 indicates the time point immediately after MMC addition. Right panels show SPβ excision (top panel) and *spsM* reconstitution (bottom panel) during sporulation. *B. subtili*s 168 cells were grown in DSM, and samples were taken at the indicated times (in h) after the onset of sporulation (T_0_). The DNA samples were digested with *Nde*I and subjected to Southern blotting. (C) Mother cell-specific SPβ excision. Chromosomal DNA from the vegetative cells (V) at T_−1_, whole sporangia (W) at T_8_, and the forespores (FS) at T_8_ were isolated, digested with *Nde*I, and subjected to Southern blotting. (D) Lytic activity of SPβ phages. SPβ phage lysate, which was prepared by treating the *B. subtilis* 168 vegetative cells with MMC, was spotted on the plate (MMC). The DSM culture of *B. subtilis* 168 at T_6_, T_12_, T_24_, and T_48_ was centrifuged and the supernatant was filtrated with 0.44 µm Millex filter (Millipore). The filtrate was spotted on the lawn of a SPβ sensitive strain CU1050 (DSM T_24_ and DSM T_48_). (E) Horizontal transfer of *spsM* rearrangement system. A new SPβ-lysogen, CU1050 (SPβ) was obtained by infecting CU1050 cells with the SPβ phage lysate. The CU1050 and CU1050 (SPβ) cells were induced to sporulate on DSM-agar plates at 37°C for 3 (Vegetative cells, Veg) and 12 hours (Sporulating cells, Spo). Chromosomal DNA of the CU1050 and CU1050 (SPβ) cells was subjected to Southern blotting.

Figure 1A shows a diagram of the 134-kb long SPβ prophage from the *B. subtilis* 168 genome. *sprA* (formerly *yokA*; NCBI gene locus tag, BSU21660), which is located immediately upstream of *ypqP*, encodes a putative site-specific DNA recombinase, which shares 26% identity with SpoIVCA of the *skin* element. The attachment sites are indicated by triangles. When *B. subtilis* 168 vegetative cells are treated with mitomycin C (MMC), SPβ is excised (Figure 1B, left panel). Specifically, a wild-type culture was grown in Luria-Bertani (LB) medium and MMC (0.5 µg/ml) was added to the medium during the early exponential phase of growth [optical density at 600 nm (OD_600_) = 0.25]. DNA samples were extracted from the cells at different time points after MMC addition and digested with *Nde*I. From 0 to 120 min after MMC treatment, Southern blotting using the *sprA*-specific probe (*sprA* probe) detected a 9.9-kb band (corresponding to the DNA arrangement before SPβ excision). In addition to the 9.9-kb band, a second 5.6-kb band was detected at 60, 90, and 120 min after MMC treatment, which indicated SPβ excision and reconstitution of *spsM*. Subsequently, to examine *spsM* rearrangement during sporulation, we performed Southern blotting using DNA samples from sporulating *B. subtilis* 168 cells (Figure 1B, right panels). The wild-type cells were cultured at 37°C in liquid Difco sporulation medium (DSM) and harvested at successive time points one hour before, at the onset of stationary phase and every hour until 8 hours after the onset of stationary phase. Southern blotting using the *sprA* probe detected the 9.9-kb band from T_−1_ to T_8_ (Figure 1B, right top panel). The 5.6-kb band was detected at T_3_ and later, thereby indicating that SPβ was excised during sporulation without the need for MMC treatment (Figure 1B, right top panel). We also examined the *spsM* rearrangement using the *ypqP*-specific probe (*ypqP* probe) (Figure 1B, right bottom panel). In addition to the 9.9-kb band, a 6.1-kb band, which corresponded to the composite *spsM*, was detected at T_3_ and later. To confirm *spsM* reconstitution, we determined the DNA sequences at the junction sites of the excised SPβ and composite *spsM*. The sequencing data showed that SPβ excision in the sporulating cells occurred at the same site as that in the MMC-treated vegetative cells (Figure S1B) [23]. The SPβ attachment sites contain 16-bp core sequences (Figure S1B, nucleotides boxed in red) and 16-bp inverted repeat sequences (Figure S1B, arrows). Next, we determined the compartment where the *spsM* rearrangement occurred, i.e., the mother cell or forespore. The mother cell DNA and forespore DNA were isolated from wild-type cells at T_8_ and subjected to Southern blotting. The 5.6-kb and 6.1-kb bands were detected only in the mother-cell compartment, which indicated that SPβ excision during sporulation was a mother cell-specific event and that the SPβ prophage DNA is maintained in the spore genome (Figure 1C). To evaluate the ability of the excised SPβ to form phage particles during sporulation, the supernatant of the DSM culture was filtered and spotted onto a lawn produced by a SPβ-sensitive strain CU1050 [27]. Plaques were not formed (Figure 1D), suggesting that SPβ excised during sporulation is not a phage particle. Nevertheless, we confirmed that the *spsM* rearrangement can occur during sporulation in a new SPβ lysogen, CU1050 (SPβ), which was obtained by infecting CU1050 cells with the SPβ phage lysate (Figure 1E). This result indicates that the *spsM* rearrangement system can be transferred to a new host via SPβ phage infection.

### DNA Rearrangement of *Bacillus amyloliquefaciens spsM*

**Figure 2:**
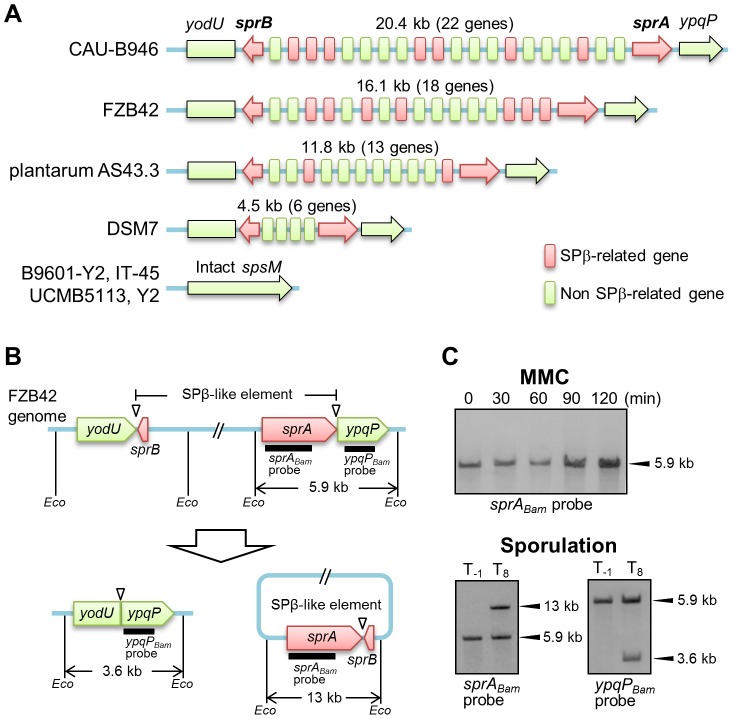
DNA rearrangement of *spsM* in *B. amyloliquefaciens*. (A) Schematic representation of the gene organization of SPβ-like elements at the *spsM* locus of *B. amyloliquefaciens* strains. Eight *B. amyloliquefaciens* strains with genome sequences deposited in KEGG are shown here as representative examples. The *yodU* and *ypqP* ORFs are located at the left and right ends, respectively. The red arrows indicate *sprA* and *sprB*, which are required for SPβ excision. The size (kb) of the element and number of genes in the element are shown above the diagram. The red and green boxes indicate SPβ-related and non-SPβ-related genes, respectively. The conserved SPβ genes in *B. amyloliquefaciens* strains are listed in Table S1. (B) Diagram of SPβ-like element excision in *B. amyloliquefaciens* FZB42. The thick lines indicate the DIG-labeled probes used for Southern blotting. *Eco* indicates *Eco*RV sites. Triangles point to the attachment sites for SPβ. (C) DNA rearrangement of *spsM* in *B. amyloliquefaciens* FZB42. *B. amyloliquefaciens* FZB42 cells were cultured at 37°C in DSM medium. Chromosomal DNA samples from the cells in the vegetative (T_−1_) and sporulation phases (T_8_) were digested with *Eco*RV and subjected to Southern blotting. The *sprA_Bam_* and *ypqP_Bam_* probes were specific to *B. amyloliquefaciens sprA* and *ypqP*, respectively.

In addition to *B. subtilis* 168, several *B. amyloliquefaciens* strains carry a prophage sequence similar to SPβ at the *spsM* locus (Figure 2A and Table S1). The numbers of SPβ-related genes varied considerably among all of these strains. These SPβ-like elements are likely to be remnants of the SPβ prophage and have probably lost their ability to form infectious phage particles, because large parts of the SPβ-related genes were missing. Since the gene encoding the putative site-specific recombinase, *sprA*, was conserved in all of these elements, we examined whether the SPβ-like element was excised from the chromosome in *B. amyloliquefaciens* strain FZB42 (BGSC catalogue number, 10A6). Figure 2B shows a diagram of the SPβ-like element in *B. amyloliquefaciens* FZB42. First, we tested whether the element responded to MMC by analyzing a DNA sample prepared from MMC-treated vegetative cells of strain FZB42 and subjected to Southern blotting. The *B. amyloliquefaciens sprA*-specific probe (*sprA_Bam_* probe) detected a single 5.9-kb band from 0 to 120 min after MMC addition, indicating that excision of the element did not occur in the MMC-treated vegetative cells (Figure 2C, upper panel). Subsequently, Southern blotting was performed using a DNA sample obtained from sporulating cells of strain FZB42 (Figure 2C, lower panels). Bands indicating excision of the element (13 kb, left panel) and the generation of the composite *spsM* (3.6 kb, right panel) were detected using the *sprA_Bam_* and *ypqP_Bam_* probes, respectively. These data indicate that the SPβ-like element of *B. amyloliquefaciens* FZB42 exhibits a behavior distinct from the *B. subtilis* SPβ, but similar to *skin*, because it did not respond to MMC treatment and was excised only during sporulation.

### *sprA* and *spsM* Expression and Regulation of SPβ Excision

**Figure 3:**
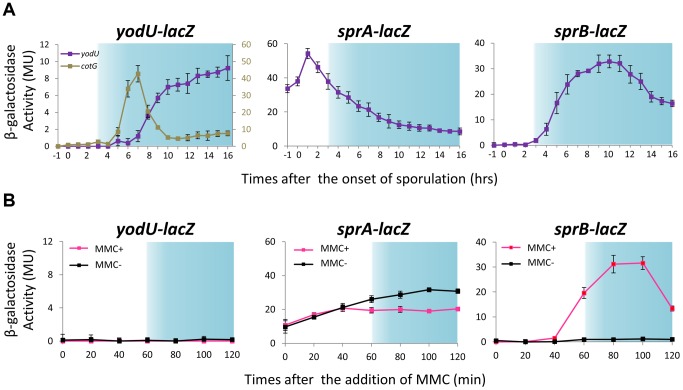
Expression of *spsM*, *sprA*, and *sprB* in response to mitomycin C treatment and during sporulation. (A) β-galactosidase activity of *B. subtilis* strains carrying *lacZ* reporter constructs during sporulation. The *B. subtilis* strains, YODUd (*yodU–lacZ*), SPRAd (*sprA–lacZ*), and BsINDB (*sprB–lacZ*), were sporulated at 37°C in liquid DSM. Aliquots were collected at various time points during sporulation, and the β-galactosidase activity (Miller units, MU) was determined using ortho-nitrophenyl-β-galactoside (ONPG) as a substrate. To compare the expression pattern of *yodU* (5′-*spsM*) to that of *cotG*, a previously-known σ^K^-dependent sporulation gene [29], the β-galactosidase activity of *cotG–lacZ* (COTGd) is shown on the left panel (gray line, right axis) along *yodU–lacZ* (purple line, left axis). SPβ excision and *spsM* rearrangement occurred at T_3_ and later time points (blue-shaded areas). The background activity was subtracted from the values. Error bars indicate ± standard deviations based on three independent experiments. (B) β-galactosidase activity of *B. subtilis* vegetative cells carrying the *lacZ* reporter construct fused transcriptionally to the promoters of *spsM*, *sprA*, and *sprB* in response to MMC treatment. The *B. subtilis* strains, YODUd (*yodU–lacZ*), SPRAd (*sprA–lacZ*), and BsINDB (*sprB–lacZ*), were cultured in liquid LB medium. MMC was added to a final concentration of 0.5 µg/ml when the cells reached an OD_600_ of 0.5. The culture was sampled at 0, 20, 40, 60, 80, 100, and 120 min after the addition of MMC. SPβ excision occurred at 60 min and later (blue-shaded areas). Error bars indicate ± standard deviations based on three independent experiments.

Considering that in *B. subtilis* SPβ excision occurs both during sporulation and in response to DNA damage, whereas in *B. amyloliquefaciens* excision of the SPβ-like element only occurs during sporulation, it is likely that different mechanisms control prophage excision during sporulation and upon MMC treatment. To analyze how SPβ controlled its excision and to determine whether *spsM* expression always followed prophage excision, we constructed transcriptional *lacZ* fusions to *yodU* (5′-*spsM*) [YODUd; *yodU*::pMutinT3, P*_yodU_*–*lacZ*] and *sprA* (SPRAd; *sprA*::pMutinT3, P*_sprA_*–*lacZ*) using the pMutinT3 insertion plasmid (Figure S2A). Insertion of the pMutinT3 vector into a genome locus causes inactivation of the corresponding gene and allows analysis of its expression profile by measuring β-galactosidase activity, because the gene of interest is now transcriptionally fused to *lacZ* [28]. In the YODUd strain, P*_yodU_*–*lacZ* was expressed during the late stages of sporulation, consistent with the previously reported σ^K^-dependency for *yodU* expression (Figure 3A, left panel, hour 8 and later). The timing of expression of *yodU* was delayed by 2 hours when compared to that of *cotG*, another σ^K^-dependent gene [29]. This delay is likely due to the fact that *yodU* expression also requires the transcription factor GerE, which regulates gene expression in the mother cell during the ultimate stage of sporulation, as previously shown [20]. By contrast, P*_yodU_*–*lacZ* was not expressed in MMC-treated vegetative cells (Figure 3B, left panel), indicating that prophage excision does not systematically trigger *spsM* expression.

**Figure 4:**
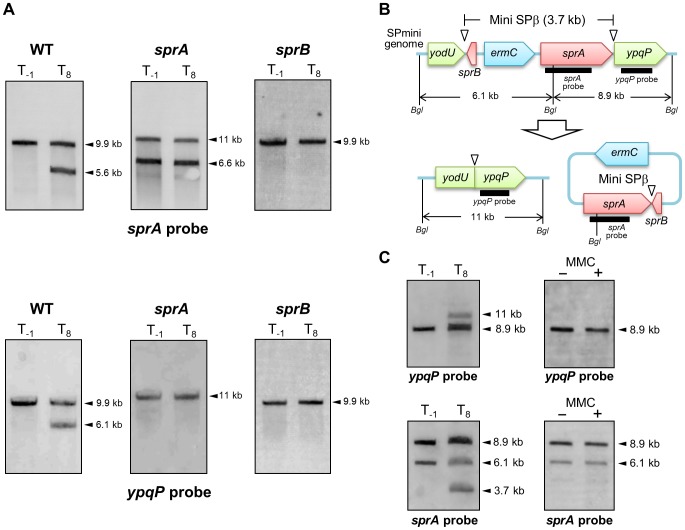
SPβ genes required for prophage excision. (A) Chromosomal DNA from the vegetative (T_−1_) and the sporulating cells (T_8_) of strain 168 (WT), SPRAd (*sprA*), and SPRBd (*sprB*) were digested with *Nde*I and subjected to Southern blotting. The genetic maps of SPRAd and SPRBd were shown in Figure S2. (B) The schematic shows construct of the SPmini strain. Thick lines indicate *sprA* and *ypqP* probes for Southern blotting. *Bgl* denotes *Bgl*II restriction sites. (C) Southern blotting. Chromosomal DNA was isolated from vegetative (left panels, T_−1_) and sporulating cells (left panels, T_8_) in the DSM culture and from the SPmini vegetative cells (OD_600_ = 0.25) grown in LB with or without MMC treatment (0.5 µg/ml) at 37°C for 60 min (right panels). DNA was digested with *Bgl*II and subjected to Southern blotting using the *sprA* and the *ypqP* probes.

Analysis of the SPRAd mutant strain by Southern blotting did not reveal any difference in the band patterns of vegetative and sporulating cells (Figures 4A, middle panels and S2A), showing that *sprA* was necessary for *spsM* reconstitution. Nicolas et al. predicted a putative binding site for the housekeeping σ factor σ^A^ at positions −85 to −57 (TTGTTT for the −35 box and TAAAAT for the −10 box) relative to the *sprA* start codon [22]. Consistent with a σ^A^-dependent pattern of expression, but at odds with a specific role for SprA during the late stages of sporulation, the P*_sprA_*–*lacZ* activity kept increasing during vegetative growth, peaked during the early stages of sporulation and gradually decreased as sporulation proceeded (Figure 3A, middle panel). The *sprA* expression level in vegetative cells was not increased by MMC addition (Figure 3B, middle panel). These unexpected results suggest that an additional factor(s) regulates the timing of prophage excision during sporulation and following DNA damage. We observed that *sprB* (formerly *yotN*; NCBI gene locus tag, BSU19820), a SPβ gene located downstream of *yodU*, was conserved in all of the SPβ-like elements (Figure 2A). It encodes a 58-aa protein with no significant similarity to characterized proteins. To test whether *sprB* was required for excision, we constructed a *sprB* deletion mutant strain (SPRBd). Southern blotting revealed that SPRBd was defective in SPβ excision (Figures 4A, right panels and S2B), indicating that *sprB* was necessary for excision. As expected, P*_sprB_*–*lacZ* was expressed during the middle and late stages of sporulation (Figure 3A, right panel) and was also induced by MMC addition to vegetative cells (Figure 3B, right panel, 60 min and later).

To examine the correlation between SPβ excision and *sprA* and *sprB* expression, we constructed the *sprA*-inducible strain (BsINDA) and the *sprB*-inducible strain (BsINDB), where *sprA* or *sprB* expression can be induced by isopropyl β-D-1-thiogalactopyranoside (IPTG) addition. SPβ was excised when *sprB* expression was induced in BsINDB, but not when *sprA* was over-expressed in BsINDA (Figure S3). Combined with the results from Figure 3, showing that P*_sprA_*–*lacZ* is expressed at significant levels during vegetative growth and the early stages of sporulation, we conclude that expression of *sprA* alone is not sufficient to excise SPβ from the chromosome (Figure S3A). By contrast, when *sprB* is induced, either in the presence of MMC, during sporulation, or artificially by IPTG addition, excision of SPβ will ensue (Figure S3B), provided that SprA is also present. In summary, both *sprA* and *sprB* are necessary for excision, but the temporal control of excision is dependent on *sprB*. To determine whether SPβ genes other than *sprA* and *sprB* were also required for excision, we constructed a SPβ mutant strain (SPmini), where all the SPβ genes were deleted, except *sprA* and *sprB*. The SPmini strain retained the capacity for *spsM* rearrangement during sporulation (Figure 4C, left panels), indicating that *sprA* and *sprB* are necessary and sufficient for SPβ excision during sporulation. By contrast, SPmini did not undergo excision upon MMC treatment (Figure 4C, right panels), suggesting that an additional gene(s) or regulatory sequence present in SPβ but absent in SPmini may be required to promote excision and/or trigger *sprB* expression following DNA damage.

### Regulation of *sprB* Expression

**Figure 5:**
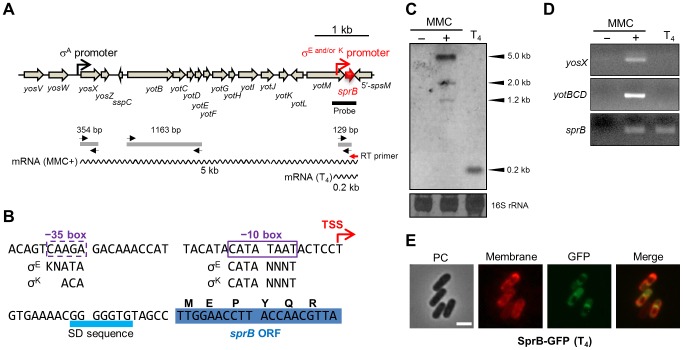
Mother cell-specific expression of *sprB* during sporulation. (A) Genetic organization of the *sprB* region. The black and red promoter symbols indicate the promoter upstream of *yosX* and the mother cell-specific promoter directly upstream of *sprB*, respectively. The thick black line indicates the *sprB* probe for Northern blotting. The wavy lines indicate the *sprB* transcripts with their respective lengths (kb). The red and black arrows indicate the *sprB*-specific primer for reverse transcription (RT primer) and the *yosX*-, *yotB*C*D*-, and *sprB*-specific primers for the PCR reactions, respectively. The gray lines show the products of RT followed by PCR amplification. (B) Nucleotide sequence of the *sprB* promoter region. The transcriptional start site (TSS) of *sprB* is indicated by the red arrow. Boxes indicate −35 and −10 elements of the *sprB* promoter. The consensus sequences for σ^E^ and σ^K^ binding are shown below (K = G or T; N = A, T, G, or T). (C) Northern blotting. Total RNA was isolated from *B. subtilis* 168 vegetative cells treated with (+) or without (−) 0.5 µg/ml MMC at 37°C for 60 min and from sporulating cells 4 hours after onset of the sporulation (T_4_). The RNA samples were subjected to Northern blotting using the *sprB* probe. The bottom panel shows methylene blue-stained 16S rRNA as a loading control. (D) RT-PCR. The *sprB* cDNA was synthesized using the *sprB*-specific primer (Figure 5A, the red arrow RT primer) and total RNA from the *B. subtilis* 168 vegetative cells treated with (+) or without (−) MMC and from sporulating cells (T_4_). Internal regions of the cDNA were amplified with the *yosX*-, *yotB*C*D*-, and *sprB*-specific primer sets. The PCR product was analyzed by 2% agarose gel electrophoresis. (E) Compartmentalization of SprB–GFP expression. BsSPRBG, carrying the *sprB*–*gfp* fusion gene under the control of the mother cell specific *sprB* promoter, was cultured at 37°C in liquid DSM containing FM4-64 (0.25 µg/ml) and kanamycin (10 µg/ml). Sporulating cells at T_4_ were observed using fluorescence microscopy. PC, phase contrast; Membrane, cell membranes stained with FM4-64; GFP, GFP fluorescence; Merge, merged images of Membrane and GFP. Scale bar, 2 µm.

Since *sprB* is a key factor in the control of SPβ excision, we analyzed its transcriptional regulation (Figure 5). We performed Northern blotting using a *sprB*-specific probe (Figure 5A, thick black line). A major band of 5.0 kb and minor bands of 1.2 and 2.0 kb were detected in MMC-treated vegetative cells but not in untreated cells (Figure 5C, columns 1 and 2). By contrast, a single 0.2-kb band was detected during sporulation (Figure 5C, column 3). This result suggested that *sprB* was transcribed from distinct promoters upon MMC treatment and during sporulation. Lazarevic et al. reported that the *yosX* gene, which is located 5 kb upstream of *sprB*, possesses a σ^A^-dependent promoter [23], but no other σ^A^-dependent promoter was predicted between *yosX* and *sprB* (Figure 5A). Thus, it is likely that the major 5.0 kb band detected upon MMC treatment corresponds to a transcript originating from the *yosX* promoter, while the minor bands could correspond to truncated transcripts. Next, we performed RT-PCR using a *sprB*-specific reverse transcription primer (Figure 5A, RT primer, red arrow) followed by PCR amplification of the *sprB* cDNA using *yosX*, *yotBCD*, or *sprB* specific primers (Figure 5A, black arrows). When the *sprB* cDNA was obtained from the MMC-treated cells (Figure 5D, column 2) the *yosX*, *yotBCD*, and *sprB* regions were successfully amplified, whereas when the *sprB* cDNA was obtained from sporulating cells, only the *sprB* region could be amplified (Figure 5D, column 3). This result indicates that *sprB* is indeed co-transcribed with the upstream genes upon MMC treatment while it appears to be monocistronically transcribed during sporulation.

To determine the 5′ end of the *sprB* transcript during sporulation, we carried out 5′ RACE PCR with total RNA extracted at T_4_. The *sprB* transcriptional start site (TSS) was found to be located 20 nt upstream of the start codon (Figures 5B and S4). Using DBTBS Search Tools (http://dbtbs.hgc.jp/) [30], a putative σ^E^- or σ^K^-binding site was found directly upstream of the TSS of *sprB* (Figure 5B). However, while the −10 element was a perfect match to the σ^E^- or σ^K^-consensus sequence, the putative −35 element of the *sprB* promoter was an imperfect match (Figure 5B). It is therefore possible that an additional mother cell transcription factor, such as SpoIIID, GerR or GerE, is required along with σ^E^ or σ^K^ for optimal expression of *sprB*. To test whether *sprB* expression is restricted to the mother cell, as would be expected if it is controlled by a σ^E^ or σ^K^, we constructed strain BsSPRBG, which harbors a plasmid carrying the translational fusion *sprB*–*gfp* without the upstream phage genes. As expected, GFP fluorescence in BsSPRBG was detected only in the mother cell (Figure 5E). Importantly, this observation is also consistent with the data presented above (Figure 3A), where P*_sprB_*–*lacZ* activity was detected during the middle to the late stages of the sporulation, when σ^E^ and σ^K^ are most active.

### Negative Staining of Spores Is Dependent on *spsM*

**Figure 6:**
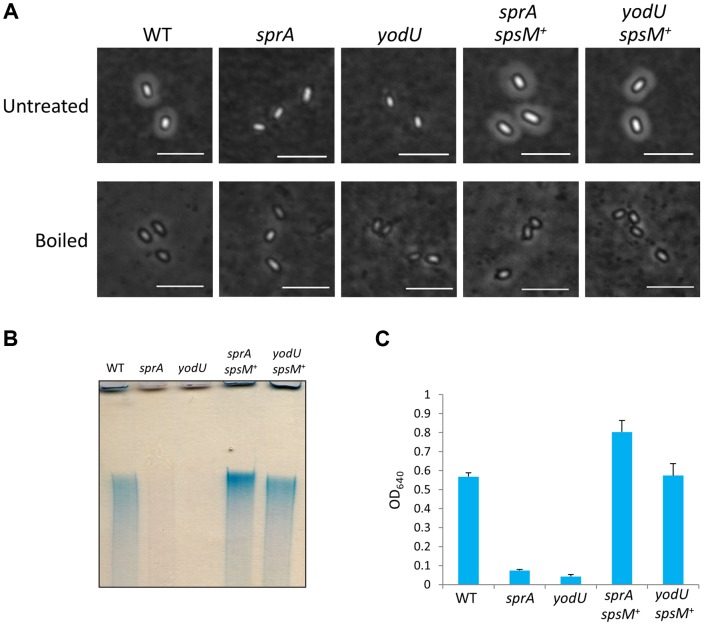
Analysis of *B. subtilis* spore surface components. (A) Negative staining with Indian ink of the *B. subtilis* wild-type and mutants. The purified spores from strain 168 (WT), SPRAd (*sprA*), YODUd (*yodU*), SPRAc (*sprA spsM* ^+^), and YODUc (*yodU spsM* ^+^) were negatively stained with Indian ink and observed using phase-contrast microscopy. Untreated, native spores; boiled, heat-treated spores at 98°C 10 min in SDS buffer. Scale bars, 4 µm. (B) Electrophoresis of *B. subtilis* spore surface extracts. Spore surface extracts from strain 168 (WT), SPRAd (*sprA*), YODUd (*yodU*), SPRAc (*sprA spsM* ^+^), and YODUc (*yodU spsM* ^+^) were loaded onto a 5% native polyacrylamide gel. The gel was stained with Stains-All after electrophoresis. (C) Quantification of the polysaccharides in spore surface extracts. The spore surface polysaccharides from *B. subtilis* spores were ethanol-precipitated. The precipitants were dissolved in water and reacted with Stains-All. The amounts of polysaccharides were determined by measuring the OD_640_ according to the method described by Hammerschmidt et al. [32]. Error bars indicate ± standard deviations based on three independent experiments.

To investigate the functional role of *spsM* in sporulation, we used the YODUd (*yodU*) and SPRAd (*sprA*) strains. Since SPRBd exhibited the same phenotype as SPRAd, only the SPRAd strain will be considered further. We analyzed the morphologies of wild-type, YODUd, and SPRAd spores using phase-contrast microscopy and a negative staining procedure. When the spores were negatively-stained with Indian ink [31], which is a stain commonly used to reveal polysaccharide capsules, a clear halo was visible around the wild-type spores, but not around the YODUd and SPRAd spores (Figure 6A, top panels). The appearance of a halo is consistent with the presence of polysaccharides around the wild-type spore. Introduction of the composite *spsM* gene at the *amyE* locus of the mutant strains complemented the *sprA* and *yodU* mutations (SPRAc and YODUc) in the sense that the halo was restored (Figure 6A, top panels, *sprA spsM* ^+^ and *yodU spsM* ^+^). In addition, we observed that this putative polysaccharide layer of the wild-type spore was loose, because it can easily be removed from the spores by boiling in a buffer containing SDS. After this treatment, the wild-type and *spsM* ^+^ spores became indistinguishable from the SPRAd and YODUd spores, as none of the spores exhibited a halo (Figure 6A, bottom panels). These results suggest that the composite *spsM* is necessary for the production of an external spore structure most likely composed of polysaccharides.

### Analysis of the Chemical Composition of the Spore Surface

Surface extracts from wild-type spores were loaded on a 5% polyacrylamide gel, separated by electrophoresis and stained with stains-All, a cationic carbocyanine dye that stains polysaccharides, nucleic acids, and acidic proteins. The spore surface component was detected as a bright blue band (Figure 6B), which indicated the presence of a high molecular weight substance. The blue band was not detected in extracts from SPRAd and YODUd (Figure 6B, *sprA* and *yodU*), whereas it was detected in extracts from SPRAc (*sprA spsM* ^+^) and YODUc (*yodU spsM^+^*). These results imply that the formation of the spore surface component is dependent on the function of the composite *spsM*. SpsM is a paralog of a polysaccharide synthesis protein, EpsC [26]. Thus, the high molecular weight substance from the spore surface is likely to be a polysaccharide, whose synthesis and/or attachment to the spore is dependent on SpsM. In addition, this high molecular weight substance was inferred to be produced in the mother cell because the composite SpsM protein fused to GFP was observed to reside in the mother cell during sporulation (Figure S5), consistent with its regulation by σ^K^ [20]. We quantified the amount of spore surface component using the method described by Hammerschmidt et al. [32]. The levels of high molecular weight substance in the SPRAd and YODUd spore surface extracts decreased to 12.5% and 5.0% of the amount isolated from wild-type spores (Figure 6C).

Next, we analyzed the monosaccharide composition of the wild-type spore surface extract. The extract was hydrolyzed and fluorescently labeled with 4-amino-benzoic acid ethyl ester (4-ABEE). HPLC analysis detected three major peaks. By comparison to fluorescently labeled monosaccharide standards, we infer that the two peaks detected in the extracts at retention times of 10.9 and 30.8 min corresponded to galactose and rhamnose, respectively (Figure S6, peaks 3 and 12). A peak at 6.4 min, which did not correspond to any monosaccharide standard, was considered to be an unknown monosaccharide(s) or could result from an incomplete hydrolysis of oligosaccharides. The galactose and rhamnose peaks accounted for 21.1% and 68.1% of total amount of monosaccharides detected by HPLC, respectively. The presence of rhamnose at the spore surface has been previously reported and was shown to be dependent on the enzymes SpsI, SpsJ, SpsK and SpsL, whose synthesis is dependent on σ^K^ during sporulation [20], [33], [34]. In conclusion, our experiments indicate that polysaccharides are present in spore surface extracts and that *spsM* is involved in their production and/or attachment to the spore envelope.

### Properties of the *spsM* Mutant Spores

**Figure 7:**
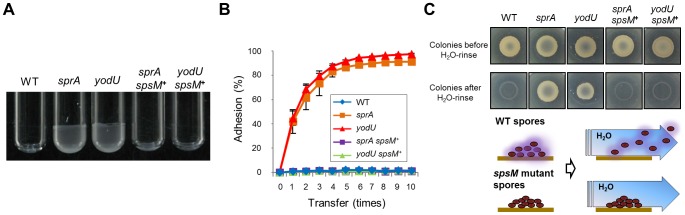
Spore properties. (A) Adhesion of the mutant and wild-type spores to glass tubes. The spores purified from strain 168 (WT), SPRAd (*sprA*), YODUd (*yodU*), SPRAc (*sprA spsM* ^+^), and YODUc (*yodU spsM* ^+^) were resuspended in water and the final OD_600_ was adjusted to 15. Each 30 µl of spore resuspension was added to a Pyrex tube (13×100 mm, Corning) and vortexed gently for 30 s. After removing the spore resuspensions, the glass tubes were briefly dried and images were acquired. (B) Adhesion of the mutant and wild-type spores to polypropylene tubes. Adhesion (%) was determined by 10 successive binding reactions of the spores to the tubes. Error bars indicate ± standard deviations based on three independent experiments. (C) The polysaccharide layer facilitates spore dispersal through water flow. Overnight cultures of *B. subtilis* cells grown in LB medium were spotted onto DSM-agar plates. The plates were incubated at 37°C for 1 week. Each colony was confirmed as containing>95% free spores using phase-contrast microscopy. The images show the spore colonies on the DSM plates before (upper panels) and after rinsing with 1 ml of DDW (lower panels). The wild-type spores on the plates were dispersed by water, whereas the mutant spores stuck to the plates.

Subsequently, we investigated the functional roles of the spore polysaccharides. YODUd and SPRAd retained the ability to produce phase-bright and wet-heat resistant spores although spore titers in DSM cultures were slightly smaller than that of the wild type (Table S2). In addition, a SPβ-cured strain, SPless, produced normal wet-heat resistant spores with a sporulation efficiency that was comparable to that of the wild type (Table S2). However, we noticed that the mutant spores exhibited significant differences in their properties. The purified mutant spores formed aggregates and displayed enhanced adhesion to solid surfaces, such as borosilicate glass and polypropylene. Figure 7A reports that mutant spores adhere to Pyrex tubes (13×100 mm, Corning), whereas wild-type and *spsM* ^+^ spores do not. Figure 7B shows the result of an adhesion test using polypropylene tubes (see Materials and Methods). While 80%–90% of YODUd and SPRAd spores had adhered to the tubes after five transfers, wild-type and *spsM^+^* spores barely adhered to the tube even after ten transfers. Finally, we investigated the adhesive properties of the mutant spores on DSM-agar plates (Figure 7C). *B. subtilis* cells were cultured at 37°C on DSM plates for a week to allow sporulation. After this period, >95% cells on the plates became mature spores (Figure 7C, upper panels). After the plate was rinsed with water, the wild-type spores dispersed in water and disappeared from the plate (Figure 7C, lower panels). However, the SPRAd and YODUd spores were barely resuspended in water, and most of the spores were left on the plates. Therefore, our results suggest that the spore polysaccharide are beneficial for the dispersal of *B. subtilis* spores through water and help prevent adhesion to certain types of surfaces.

## Discussion

**Figure 8:**
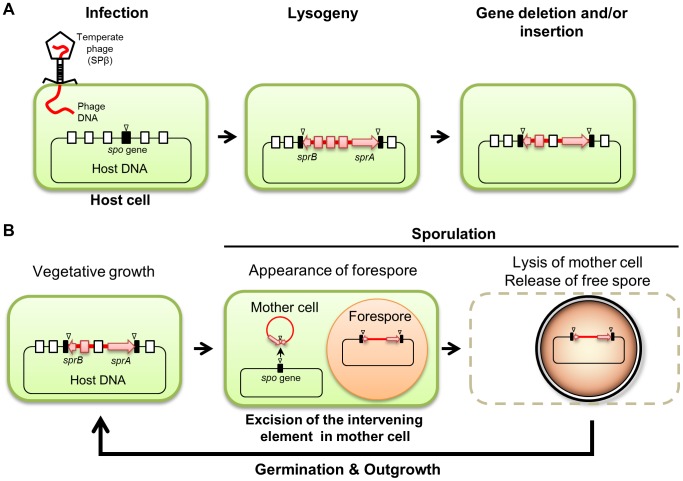
Model of the phage-mediated DNA rearrangement. (A) A model of the sporulation-specific phage-mediated gene rearrangement, based on the cases of SPβ in *B. subtilis* and *B. amyloliquefaciens*. (B) Maintenance of the intervening element in the host genome. Sporulation gene (*spo* gene), black box; attachment sites, triangle; intervening element, red line; *sprA* and *sprB*, red arrow; phage-related genes, red box; host genes, open box.

We demonstrated that both *B. subtilis* and *B. amyloliquefaciens* reconstitute a functional *spsM* gene during sporulation through developmentally-controlled excision of the SPβ prophage (Figures 1 and 2); however, while SPβ is an active prophage in *B. subtilis*, it has become a cryptic prophage in strains of *B. amyloliquefaciens* (Figure 2 and Table S1). The observation that the *spsM* rearrangement system can be transferred to a non-lysogenic strain via SPβ infection (Figure 1E) suggests that the element was originally acquired by the current lysogenic strains following an infection with an ancestral phage identical or very closely related to SPβ. We speculate that the strains of *B. amyloliquefaciens* have been infected with SPβ earlier than *B. subtilis* and have since lost most of the original phage genes, probably because they did not confer significant advantages or may even be harmful to the host (Figure 8A).

Prophage-mediated reconstitution of mother cell-specific sporulation genes is a common event since other intervening elements (e.g., *skin*, *vfbin*, and *vrin*) that carry phage-related genes have been previously observed in several spore-forming bacteria [18], [35]. Similar to *B. amyloliquefaciens* SPβ, these elements are the descendants of ancestral prophages and have now become defective for producing phage particles, but are still being excised under specific conditions (Figure 8A). Importantly, the excision of these elements from the host genome is developmentally regulated and confined to a terminally differentiated cell type, the mother cell (Figure 1C) [6], [16], [18]. Limiting the DNA rearrangement to the mother cell genome ensures that the phage DNA is maintained in the spore genome (Figure 8B). Thus, after spore germination, SPβ is vertically transferred to the progeny upon cell division as a permanent element in the host genome sequence.

Insertion of prophages in sporulation genes is advantageous to the host for at least two reasons: (1) to add one level of control to the progression of sporulation; and (2) to acquire immunity against other phages. As an example of the first type of benefit, the presence of *skin* in the host genome was shown to be required for efficient sporulation in *Clostridium difficile* [17], even though it is dispensable in *B. subtilis* [36]. During sporulation in *B. subtilis*, the temporal control of σ^K^ activity is achieved by triggering the proteolytic removal of an inhibitory pro-sequence at its N-terminus [37]. Since σ^K^ does not possess the pro-sequence in *C. difficile*, another regulatory mechanism is required to control the timing of σ^K^ activation [17]. Regarding the second type of benefit, phages constitute an ideal vehicle for the host to acquire genes that provide selective advantages, especially as protection against other phage infections. In addition, sporulation genes are suitable locations for bacterial attachment sites because they are not essential for vegetative cell growth and viability. In general, lysogenic bacteria become immune to further infections by acquiring the ability to synthesize repressor proteins for closely related phages. Furthermore, in the case of SPβ, the prophage carries both *sunA*, which encodes sublancin, an antimicrobial that inhibits cell growth of non-SPβ lysogens [38], and *nonA*, which confers resistance to infections by the virulent phage SP10 [39]–[41]. Since the SPβ-cured strain, SPless, produces normal spores (Table S2), the presence of SPβ in the *B. subtilis* genome is more likely to be beneficial to the host by providing immunity against other phages rather than adding a layer of control to sporulation progression. Recently, Rabinovich et al. have reported a similar prophage excision event in *Listeria monocytogenes* [42]. In this case, a functional *comK* gene is reconstituted to favor escape from phagocytosis. This observation suggests that prophage-mediated gene reconstitution is common among bacteria and is not limited to spore-formers.

Of the SPβ genes, we found that only *sprA* and *sprB* were required for excision (Figure 4). We have shown that *sprB* was expressed in response to MMC treatment (DNA damage) and is developmentally regulated during sporulation, whereas *sprA* was expressed irrespective of the host cell status (Figure 3). Moreover, overexpression of *sprB*, but not of *sprA*, successfully promoted *spsM* reconstitution in vegetative cells, even without MMC induction (Figure S3). Our results suggest that *sprB* is the factor that controls the timing of SPβ excision. SprA belongs to a family of large serine recombinases, which rely on recombination directionality factors (RDFs) to promote excision [43]. RDFs are small DNA-binding proteins that initiate the assembly of the recombinase–DNA complexes. SprB may serve as a RDF for the SprA recombinase during SPβ excision. Lazarevic et al. found putative SPβ repressor-binding sites called SPBRE in the promoter regions of *yorE*, *yorM*, *yorZ*, and *yosX* [23]. Repression is expected to be relieved upon MMC treatment. Thus, activation of *sprB* expression in response to DNA damage seems to result from derepression of *yosX* and its downstream genes, which include *sprB*. Importantly, SPmini is not subjected to excision upon MMC treatment (Figure 4C), since it is lacking the SOS-inducible phage genes upstream of *sprB*. In addition, we identified a mother cell-specific promoter immediately upstream of the *sprB* gene (Figures 5AB). We propose that the reason why phage particles are not produced after SPβ excision during sporulation is because many SPβ genes lack sporulation-specific promoters, resulting in insufficient production of phage structural components. After SPβ excision, transcription of *spsM* is controlled by σ^K^ [20]. The σ^K^-encoding gene, *sigK*, is itself generated by excision of *skin* [6]. Therefore, expression of the composite *spsM* requires two DNA rearrangement events mediated by the phage elements SPβ and *skin*.

Our study also revealed an important connection between *spsM* function and *B. subtilis* spore surface properties. In *Bacillus anthracis* and *Bacillus cereus* strains, spores are surrounded by an exosporium, which is a loose-fitting and balloon-like structure, containing glycoproteins and polysaccharides [44], [45]. The exosporium is not observed in *B. subtilis* spores, but the crust can be considered to be an exosporium-like structure, even though it does not display the balloon-like structure of a typical exosporium. The protein composition of the crust has been characterized to some extent, in the sense that the coat proteins CgeA, CotG, and CotXYZ were identified as crust components [9], [10]; however, beyond the fact that rhamnose, whose synthesis is dependent on *spsIJKL* [34], is a component of the spore surface [33], the spore polysaccharide composition in *B. subtilis* remains poorly characterized. Our analyses indicate that the *B. subtilis* spore polysaccharide also comprises galactose in addition to rhamnose and possibly another monosaccharide of unknown identity. In addition, we have shown that the production and/or attachment of the polysaccharide to the spore surface were *spsM*-dependent (Figure 6). Our results also indicated that although *spsM* mutant spores were as heat-resistant as wild type spores (Table S2), they were considerably more sticky and aggregated in water (Figure 7). The slight reduction of the spore numbers in the *spsM* mutant strains (Table S2) may be due to their increased adhesive properties. The hydrophobic phenotype of the *spsM* mutant spores may be attributable to the hydrophobic nature of the crust proteins CotXYZ and CgeA proteins [46]. In the absence of polysaccharide addition, these proteins become directly exposed at the spore surface, and the consequence may be a decrease in the solubility of spores in water. In natural environments, water flow, such as rainfall, rivers, and sea currents, is likely to play a role in spore dispersal. For an immotile spore, the ability to be transported to a different niche, where it can germinate and resume growth, constitutes a major advantage.

In conclusion, *B. subtilis* SPβ prophage has two pathways to excision. In response to host DNA damage, the SPβ prophage is excised from the host genome to form phage particles. By contrast, during sporulation, SPβ excision occurs in the mother cell to reconstitute a sporulation gene, *spsM*, a necessary event for spore polysaccharide synthesis. Although phage particle formation does not occur during sporulation, the SPβ prophage is propagated vertically to the progeny because phage excision is limited to the mother cell genome.

## Materials and Methods

### Bacterial Strains and Genetic Manipulations

The primers used in this study are shown in Table S3. The bacterial strains and plasmids used in this study are listed in Table S4. Standard genetic manipulations of *B. subtilis* were performed as previously described [47].

### Strain Construction

Internal segments of *yodU* (+28 to +244 relative to the first nucleotide of the start codon), *sprA* (+29 to +990), and *cotG* (+21 to +217) were amplified from the chromosome of *B. subtilis* 168 using primer pairs P01/P02, P03/P04, and P05/P06, respectively. PCR products were digested with *Hin*dIII and *Bam*HI, and inserted into the *Hin*dIII–*Bam*HI site of pMutinT3. The resulting pMUT-yodU, pMUT-sprA, and pMUT-cotG, plasmids were introduced into *B. subtilis* 168-competent cells to disrupt *yodU*, *sprA*, and *cotG*, respectively. The resulting YODUd, SPRAd, and COTGd strains were selected on Luria-Bertani (LB) agar plates containing 0.3 µg/ml erythromycin.

To construct BsINDA and BsINDB, the 5′ portions containing the SD sequence of *sprA* (−27 to +990) and of *sprB* (−20 to +89) were amplified using primer pairs P07/P04 and P08/P09, respectively. PCR products were digested with *Hin*dIII and *Bam*HI and inserted into the *Hin*dIII–*Bam*HI site of pMutinT3, which allows generation of a fusion transcript with a gene encoding β-galactosidase and placing genes downstream of an IPTG-inducible promoter (P_spac_). The resulting plasmids, pMUT-sprAind and pMUT-sprBind, were introduced into *B. subtilis* 168-competent cells. The transformants were selected on LB-agar plates containing 0.3 µg/ml erythromycin.

To obtain the SPβ-cured strain (SPless), we cultivated BsINDB at 37°C in LB liquid medium in the presence of 0.5 mM IPTG overnight. The culture was spread on a LB-agar plate after dilution with fresh LB medium. The plate was incubated at 37°C overnight. The next day, SPβ-cured colonies were selected by colony PCR using primer pair P10/P11 and by erythromycin sensitivity.

A *sprB*-deletion mutant (SPRBd) and a strain harboring the minimized SPβ (SPmini) were constructed by double-crossing over recombination using the *ermC* gene cassettes. To construct SPRBd, DNA fragments corresponding to the upstream (−1126 to −1) and the downstream (+169 to +2246) flanking regions of *sprB* were amplified from the *B. subtlis* 168 genome using primer pairs P12/P13 and P14/P15. A DNA fragment containing the *ermC* gene was amplified from a pUCE191 plasmid vector using primer pair P16/P17. The DNA fragments were combined by over-extension PCR (OE-PCR) using the primer set P12/P15. The resulting PCR product was introduced into *B. subtilis*-168 competent cells and by double crossing-over replacement of the *sprB* locus by the *ermC* cassette. The transformants were selected on the LB-agar plates containing 0.3 µg/ml erythromycin.

For construction of the SPmini strain, the primer sets P18/P19 and P20/P04 were used for amplification of the DNA fragments containing the *sprB* gene (−331 to +1301 relative to the first nucleotide of the *sprB* start codon) and the *sprA* gene (−84 to +990 relative to the first nucleotide of the *sprA* start codon) with their promoter regions and the attachment sites. The DNA fragments were combined with the *ermC* cassette by OE-PCR with the primer set P19/P04 and used for transformation of *B. subtilis* 168. Transformants were selected on erythromycin-containing LB plates.

### Gene Complementation

DNA fragments containing a composite *spsM* with its promoter region (−374 to +1080, relative to the first nucleotide of the start codon of the composite *spsM*) were amplified from chromosomal DNA of *B. subtilis* 168 sporulating cells using primer pair P21/P22. The PCR product was digested with *Eco*RI and *Bgl*II, and inserted into the *Eco*RI–*Bam*HI site of the integration vector pMF20 [48]. The resulting plasmid pMFspsM was linearized by *Bgl*II-digestion and subsequently integrated into *amyE* locus of YODUd and SPRAd by double crossover recombination. The resulting YODUc and SPRAc strains were selected on LB agar plates containing 0.3 µg/ml erythromycin and 5 µg/ml chloramphenicol.

### Sporulation of *B. subtilis*

Overnight cultures of *B. subtilis* strains grown at 37°C in liquid LB medium were diluted 1∶100 with fresh liquid Difco sporulation medium (DSM) and incubated at 37°C with shaking. The CU1050 derivatives did not sporulate well in liquid DSM. Therefore, these strains were induced to sporulate on DSM-agar plates. One hundred microliter of overnight cultures of the CU1050 derivatives in LB medium were spread on 90-mm DSM-agar plates and incubated at 37°C.

### Isolation of Genomic DNA from *B. subtilis* and *B. amyloliquefaciens*

*B. subtilis* and *B. amyloliquefaciens* strains were cultured at 37°C in liquid DSM. We harvested 4 ml of the culture by centrifugation at various time points during sporulation. For induction of sporulation of the CU1050 derivatives, cells were spread on DSM-agar plates and incubated at 37°C for either 3 (vegetative phase) or 12 hrs (sporulation phase). Cell morphology was monitored by phase-contrast microscopy. After addition of 20 ml of deionized distilled water (DDW) to the plates, the cells were gently scraped from the plates and harvested by centrifugation. Genomic DNA was extracted as follows: cell pellets were suspended in 500 µl of TEN buffer [10 mM Tris-HCl (pH 7.5), 10 mM EDTA, and 0.1 M NaCl] containing 250 µg/ml lysozyme and 10 µg/ml RNase A. The suspension was incubated at 37°C for 20 min, supplemented with 0.1% of sodium dodecyl sulfate (SDS), and incubation was continued for 5 min. Genomic DNA was isolated by phenol extraction and precipitated by ethanol. The DNA pellet was resolved in TE buffer [10 mM Tris-HCl (pH 8.0) and 1 mM EDTA].

To isolate *B. subtilis* forespore DNA, 50 ml of the DSM culture at T_8_ were harvested by centrifugation. The cell pellets were resuspended in TEN buffer containing 250 µg/ml lysozyme and 100 µg/ml DNase I and incubated at 37°C for 20 min to lyse the mother cells and non-sporulating cells. The suspension was centrifuged and the pellet washed five times by resuspension and recentrifugation in 2 ml of TEN buffer. The forespore pellet was resuspended in SUTD buffer [1% (w/v) SDS, 8 M Urea, 50 mM Tris-HCl (pH 8.0), and 50 mM dithiothreitol] [47], [49] and incubated at 37°C for 90 min. The suspension was washed five times in 2 ml of TEN buffer. The forespore pellet was lysed with 250 µg/ml lysozyme, followed by phenol extraction and ethanol precipitation. The spore DNA pellet was resuspended in TE buffer.

### Preparation of Digoxigenin (DIG)-Labeled Probes

To prepare the DIG-labeled probes, DNA fragments corresponding to the 358-bp *ypqP* probe, the 982-bp *sprA* probe, and the 535-bp *sprB* probe were amplified from the chromosomal DNA of *B. subtilis* using the primer pairs P23/P24, P03/P04, and P18/P25, respectively. DNA fragments corresponding to the 600-bp *sprA_Bam_* and the 500-bp *ypqP_Bam_* were amplified from the chromosomal DNA of *B. amyloliquefaciens* FZB42 using the primer pairs P26/P27 and P28/P29, respectively. The resulting PCR products were gel-purified and labeled using DIG-High Prime (Roche) according to the supplier's instructions.

### Southern Blotting

Chromosomal DNA (2.5 µg) was digested with 20 U of restriction enzymes at 37°C for 16 hours, separated by 0.8% agarose gel electrophoresis and blotted onto a Hybond-N^+^ membrane (GE Healthcare) using Alkaline solution (10× SSC and 0.2 N NaOH). Hybridization and detection were performed according to the DIG Application Manual (Roche). Signals were detected by a nitro-blue tetrazolium/5-bromo-4-chloro-3-indolyl-phosphate (NBT/BCIP) reaction using the DIG Nucleic Acid Detection Kit (Roche).

### β-galactosidase Assay

YODUd (*yodU*::pMutinT3, P*_yodU_*–*lacZ*), SPRAd (*sprA*::pMutinT3, P*_sprA_*–*lacZ*), and BsINDB (*sprB*::pMutinT3, P*_sprB_*–*lacZ*, P_spac_–*sprB*) were used to monitor the *yodU*, *sprA*, and *sprB* promoter activities, respectively. The *B. subtilis* strains were sporulated at 37°C in liquid DSM. The samples were collected at various time points after the end of the exponential phase of growth. β-galactosidase activity was determined using the method described by Miller [50].

### Northern blotting

*B. subtilis* 168 cells were grown at 37°C in 50 ml LB medium up to the early log phase (OD_600_ = 0.25). The culture was further incubated at 37°C for 60 min in the presence or absence of MMC (0.5 µg/ml), and harvested by centrifugation. For preparation of the sporulating cells, the *B. subtilis* 168 cells were cultured at 37°C in 50 ml of liquid DSM and harvested at T_4_ by centrifugation. Total RNA was isolated as described previously [51]. Five micrograms of total RNA were mixed with two volumes of denaturing buffer [50% formamide, 6% formaldehyde, 20 mM morpholinopropanesulfonic acid (MOPS) (pH 7.0), 5 mM sodium acetate, 1 mM EDTA, 0.05% bromophenol blue, and 10% glycerol] and incubated at 55°C for 10 min. The denatured RNA sample was loaded to a 2% denaturing agarose gel containing 2% formaldehyde, separated by electrophoresis in 1× MOPS buffer [20 mM MOPS (pH 7.0) and 5 mM sodium acetate], and capillary-transferred to Hybond N^+^ membrane (GE Healthcare) overnight in 10× SSC buffer [1.5 M NaCl and 150 mM sodium citrate (pH 7.0)]. The resulting membrane was baked at 80°C for 2 hrs and stained with a methylene blue solution [0.03% methylene blue and 0.3 M sodium acetate (pH 5.6)]. Hybridization and detection were performed according to the DIG Application Manual (Roche). Signals were detected using CDP-Star (Roche).

### RT-PCR

Total RNA from *B. subtilis* 168 vegetative cells with or without MMC treatment and sporulation cells at T_4_ were prepared as described above. The *sprB* cDNA was synthesized from 5 µg of the total RNA by an AMV reverse transcriptase XL (Takara) using the *sprB*-specific primer P30, according to the manufacturer's instructions. Internal segments of the *sprB*, *yosX*, and *yotBCD* coding regions were amplified from the cDNA by 25-cycled PCR reactions using ExTaq (Takara) and the primer sets P08/P09, P31/P32, and P33/P34, respectively. PCR products were analyzed by 2% agarose gel electrophoresis.

### Construction of a *B. subtilis* Strain Expressing SprB-GFP

To construct the pUBsprBgfp plasmid carrying the *sprB* gene translationally fused to *gfp*, a DNA fragment containing *sprB* ORF and its sporulation-specific promoter was amplified from the *B. subtilis* 168 chromosomal DNA using primers P18/P25. An 858-bp DNA fragment of *gfp* was amplified from the pMF20 vector [48] using primer pair P35/P36. The pUB110 plasmid vector [52] was linearized by PCR using the primer set P37/P38. The *sprB* DNA fragment, the *gfp* DNA fragment and the linearized pUB plasmid were combined by OE-PCR and amplified using the primer set P18/P37. The PCR product was self-ligated with T4 DNA ligase (Takara) in the presence of polynucleotide kinase (Takara) and introduced into *B. subtilis* 168-competent cells as described previously [18]. The transformants were selected by addition of 10 µg/ml kanamycin on LB-agar plates.

### Fluorescence Microscopy

*B. subtilis* strains, BsSPRBG and BsSPSMG, were cultured at 37°C in liquid DSM containing FM4-64 (0.25 µg/ml). For the cultivation of the BsSPRBG strain carrying the pUBsprBgfp plasmid, kanamycin was added to the medium at a final concentration of 10 µg/ml. Sporulating cells were observed using fluorescence microscopy as previously described [53].

### Preparation of *B. subtilis* Spores

Overnight cultures of *B. subtilis* strains in LB medium were spread on 90-mm DSM-agar plates. The DSM plates were incubated at 37°C for 6 days and kept at 4°C for a day. After the addition of 20 ml of DDW to the plates, the spores were gently scraped from the plates. The spores were centrifuged at 8,000× *g* for 30 min. The spore pellets were resuspended in 20 ml of DDW and kept overnight at room temperature. The spores were further purified as described by Carrera et al. [54].

### Indian Ink Staining

*B. subtilis* spores were negatively stained with Indian ink, as previously described [31] with a slight modification. The purified spores were resuspended in DDW, and 2 µl of the suspension was mixed with an equivalent volume of Indian ink (Daiso Sogyo, Japan) on a slide glass. A cover glass was placed on the slide glass and any excess fluid was pushed out using thumb pressure. The negatively stained outermost layer of the spore was observed using phase-contrast microscopy.

### Preparation and Detection of Spore Surface Extracts

The purified spores were resuspended in DDW and the final OD_600_ was adjusted to 50. Next, 100 µl of the spore resuspension were added to 100 µl of the SDS buffer [125 mM Tris–HCl (pH 6.8), 2% SDS, and 4% β-mercaptoethanol] and incubated at 98°C for 10 min. The supernatant was collected after centrifugation at 20,400× *g* for 5 min and 40 µl of the supernatant was loaded onto a 5% native polyacrylamide gel, which was separated by electrophoresis at 100 V for 30 min in 1× TBE buffer [44.5 mM Tris, 44.5 mM borate, and 1 mM EDTA (pH 8.0)]. The resulting gel was stained overnight at room temperature using dye solution [0.025% (w/v) Stains-All (Sigma-Aldrich), 7.5% formamide, 3% acetic acid, and 25% 2-propanol]. The polysaccharide components in the spore surface extract were quantified as described by Hammerschmidt et al. [32]. The monosaccharide composition of the spore polysaccharide was determined as described in Supporting text S1.

### Adhesion Assay

The purified *B. subtilis* spores were resuspended in DDW, and the final OD_600_ was adjusted to 0.5. Each 1 ml of the spore resuspensions was added to a polypropylene tube (8.8×40 mm; Safe-Lock tube 2.0 ml; Eppendorf). The spore resuspension was vortexed gently for 10 s and transferred to a fresh polypropylene tube. This operation was repeated 10 times. Total amount of the spores bound to the tubes [Adhesion (%)] was calculated from the percentage decrease in OD_600_ of the spore resuspension as follows: 100×[OD*_i_*−OD*_n_*]/OD*_i_*, where OD*_i_* and OD*_n_* are the initial OD_600_ ( = 0.5) and OD_600_ of each binding reaction, respectively.

## Supporting Information

Figure S1 Amino-acid sequence alignment of SpsM proteins. (A) Multiple alignments of SpsM proteins. Amino-acids sequences of YodU, YpqP, and SpsM proteins are shown. The over-lapped amino-acids sequences between YodU and YpqP of *B. subtilis* 168 are boxed in red. Bs168, *B. subtilis* 168 (YodU, NCBI locus tagBSU19810; YpqP, BSU21670); BsBEST195, *B. subtilis* BEST195 (SpsM, BSNT03232); BaFZB42, *B. amyloliquefaciens* FZB42 (YodU, RBAM019650; YpqP, RBAM019840); BaY2, *B. amyloliquefaciens* Y2 (SpsM, MUS2345). (B) Nucleotides sequences of the joint site of the composite *spsM* gene. Nucleotide sequences of the attachment sites of SPβ and the joint site of *spsM* of *B. subtilis* 168 before and after the DNA rearrangement during sporulation were determined. The 16-bp inverted repeat sequences were indicated by arrows. The sequences boxed in red are core sequences.
(TIF)

Figure S2 Strain constructs. Schematic drawing showing the *yodU*, *sprA*, and *cotG* gene disruptions by pMutinT3 [upper line: *yodU* (YODUd); middle line: *sprA* (SPRAd); bottom line: *cotG* (COTGd)] (A), and the *sprB* gene deletion by the *ermC* cassette (SPRBd) (B). Thick lines indicate the *sprA* and *ypqP* probes for Southern blotting. *Nde* denotes *Nde*I restriction sites.
(TIF)

Figure S3 Effects of overexpression of *sprA* and *sprB* on the SPβ excision. A schematic above shows the construct of BsINDA (A) and BsINDB (B). The *B. subtilis* strains carrying the constructs of P_spac_–*sprA* (BsINDA) and P_spac_–*sprB* (BsINDB) were cultured at 37°C in LB medium. IPTG (0.2 mM) was added to the medium when the cells reached mid-log phase of cell growth (OD_600_ = 0.5). DNA was extracted from the cells at various time points after addition of IPTG and digested with *Nde*I and subjected to Southern blot using the *ypqP* probe.
(TIF)

Figure S4 Determination of the transcriptional start site of *sprB*. 5′ RACE was performed using total RNA from *B. subtilis* 168 sporulating cells (T_4_) and the *sprB*-specific primers. The sequence of the 5′ end of the *sprB* cDNA is shown. Nucleotides boxed in blue indicate the protein-coding region. The transcriptional start site (TSS) is shown as the nucleotide boxed in red. The black arrow denotes the primer sequence used for the reverse transcription reaction. The predicted *sprB* promoter region is shown in Figure 5B.
(TIF)

Figure S5 Compartmentalization of SpsM–GFP expression. The *B. subtilis* strain carrying *ypqP–gfp* (BsSPSMG) was induced to sporulate at 37°C in liquid DSM containing FM4-64 (0.25 µg/ml). The sporulating cells at T_8_ were harvested and observed by phase-contrast microscopy. PC, phase-contrast; Membrane, cell membranes stained with FM4-64; GFP, SpsM*–*GFP; Merge, merged image of Membrane and GFP. Scale bar, 2 µm.
(TIF)

Figure S6 HPLC analysis of the monosaccharide composition of the spore surface polysaccharides. The spore surface polysaccharides from *B. subtilis* strain 168 spores were hydrolyzed, ABEE-labeled, and loaded onto HPLC. The upper and lower panels show the elution profiles of the standard sugars and the samples, respectively. The *x*-axis and *y*-axis indicate the retention time (min) and fluorescence intensity (Ex 305_nm_, Em 360_nm_), respectively. The peaks of the standard sugars are indicated by triangles: 1, glucuronic acid; 2, galacturonic acid; 3, galactose; 4, mannose; 5, glucose; 6, arabinose; 7, ribose; 8, N-acetyl-mannosamine; 9, xylose; 10, N-acetyl-glucosamine; 11, fucose; 12, rhamnose; and 13, N-acetyl-galactosamine. We used 29.6 pmol of the standard sugars in the HPLC analysis, except for glucuronic acid and galacturonic acid, i.e., 148.1 pmol of glucuronic acid and galacturonic acid were used for HPLC.
(TIF)

Table S1 Genes in *B. amyloliquefaciens* SPβ-like elements.
(DOCX)

Table S2 Sporulation frequencies of *B. subtilis* strains.
(DOCX)

Table S3 Primers used in this study.
(DOCX)

Table S4 Strains and plasmids used in this study.
(DOCX)

Text S1 Supporting materials and methods and references.
(DOCX)

## References

[1] Tonegawa S (1983) Somatic generation of antibody diversity. Nature 302: 575–581 10.1038/302575a0 6300689

[2] Golden JW, Robinson SJ, Haselkorn R (1985) Rearrangement of nitrogen fixation genes during heterocyst differentiation in the cyanobacterium Anabaena. Nature 314: 419–423 10.1038/314419a0 3920531

[3] Golden JW, Mulligan ME, Haselkorn R (1987) Different recombination site specificity of two developmentally regulated genome rearrangements. Nature 327: 526–529 10.1038/327526a0 3035382

[4] Carrasco CD, Buettner JA, Golden JW (1995) Programmed DNA rearrangement of a cyanobacterial hupL gene in heterocysts. Proc Natl Acad Sci USA 92: 791–795 10.1073/pnas.92.3.791 7846053 PMC42706

[5] Ramaswamy KS, Carrasco CD, Fatma T, Golden JW (1997) Cell-type specificity of the Anabaena fdxN-element rearrangement requires xisH and xisI. Mol Microbiol 23: 1241–1249 10.1046/j.1365-2958.1997.3081671.x 9106215

[6] Stragier P, Kunkel B, Kroos L, Losick R (1989) Chromosomal rearrangement generating a composite gene for a developmental transcription factor. Science 243: 507–512 10.1126/science.2536191 2536191

[7] Driks A (2004) The Bacillus spore coat. Phytopathology 94: 1249–1251 10.1094/PHYTO.2004.94.11.1249 18944462

[8] McKenney PT, Driks A, Eichenberger P (2013) The Bacillus subtilis endospore: assembly and functions of the multilayered coat. Nat Rev Microbiol 11: 33–44 10.1038/nrmicro2921 23202530 PMC9910062

[9] McKenney PT, Driks A, Eskandarian HA, Grabowski P, Guberman J, et al (2010) A distance-weighted interaction map reveals a previously uncharacterized layer of the Bacillus subtilis spore coat. Curr Biol 20: 934–938 10.1016/j.cub.2010.03.060 20451384 PMC2920530

[10] Imamura D, Kuwana R, Takamatsu H, Watabe K (2011) Proteins involved in formation of the outermost layer of Bacillus subtilis spores. J Bacteriol 193: 4075–4080 10.1128/JB.05310-11 21665972 PMC3147665

[11] Losick R, Stragier P (1992) Crisscross regulation of cell-type-specific gene expression during development in B. subtilis. Nature 355: 601–604 10.1038/355601a0 1538747

[12] Rudner DZ, Losick R (2001) Morphological coupling in development: lessons from prokaryotes. Dev Cell 1: 733–742 10.1016/S1534-5807(01)00094-6 11740935

[13] Higgins D, Dworkin J (2012) Recent progress in Bacillus subtilis sporulation. FEMS Microbiol Rev 36: 131–148 10.1111/j.1574-6976.2011.00310.x 22091839 PMC3237856

[14] Sato T, Samori Y, Kobayashi Y (1990) The cisA cistron of Bacillus subtilis sporulation gene spoIVC encodes a protein homologous to a site-specific recombinase. J Bacteriol 172: 1092–1098. 2105293 10.1128/jb.172.2.1092-1098.1990 PMC208541

[15] Popham DL, Stragier P (1992) Binding of the Bacillus subtilis spoIVCA product to the recombination sites of the element interrupting the σ^K^-encoding gene. Proc Natl Acad Sci USA 89: 5991–5995 10.1073/pnas.89.13.5991 1631085 PMC402124

[16] Sato T, Harada K, Ohta Y, Kobayashi Y (1994) Expression of the Bacillus subtilis spoIVCA gene, which encodes a site-specific recombinase, depends on the spoIIGB product. J Bacteriol 176: 935–937. 8300549 10.1128/jb.176.3.935-937.1994 PMC205134

[17] Haraldsen JD, Sonenshein AL (2003) Efficient sporulation in Clostridium difficile requires disruption of the σ^K^ gene. Mol Microbiol 48: 811–821 10.1046/j.1365-2958.2003.03471.x 12694623

[18] Abe K, Yoshinari A, Aoyagi T, Hirota Y, Iwamoto K, et al (2013) Regulated DNA rearrangement during sporulation in Bacillus weihenstephanensis KBAB4. Mol Microbiol 90: 415–427 10.1111/mmi.12375 24015831

[19] Kunst F, Ogasawara N, Moszer I, Albertini AM, Alloni G, et al (1997) The complete genome sequence of the gram-positive bacterium Bacillus subtilis. Nature 390: 249–256 10.1038/36786 9384377

[20] Eichenberger P, Fujita M, Jensen ST, Conlon EM, Rudner DZ, et al (2004) The program of gene transcription for a single differentiating cell type during sporulation in Bacillus subtilis. PLoS Biol 2: e328 10.1371/journal.pbio.0020328 15383836 PMC517825

[21] Steil L, Serrano M, Henriques AO, Völker U (2005) Genome-wide analysis of temporally regulated and compartment-specific gene expression in sporulating cells of Bacillus subtilis. Microbiology 151: 399–420 10.1099/mic.0.27493-0 15699190

[22] Nicolas P, Mäder U, Dervyn E, Rochat T, Leduc A, et al (2012) Condition-dependent transcriptome reveals high-level regulatory architecture in Bacillus subtilis. Science 335: 1103–1106 10.1126/science.1206848 22383849

[23] Lazarevic V, Düsterhöft A, Soldo B, Hilbert H, Mauël C, et al (1999) Nucleotide sequence of the Bacillus subtilis temperate bacteriophage SPβc2. Microbiology 145: 1055–1067 10.1099/13500872-145-5-1055 10376821

[24] Lin WS, Cunneen T, Lee CY (1994) Sequence analysis and molecular characterization of genes required for the biosynthesis of type 1 capsular polysachcaride in Staphylococcus aureus. J Bacteriol 176: 7005–7016. 7961465 10.1128/jb.176.22.7005-7016.1994 PMC197074

[25] Fry BN, Korolik V, ten Brinke JA, Pennings MT, Zalm R, et al (1998) The lipopolysaccharide biosynthesis locus of Campylobacter jejuni 81116. Microbiology 144: 2049–2061 10.1099/00221287-144-8-2049 9720026

[26] McLoon AL, Guttenplan SB, Kearns DB, Kolter R, Losick R (2011) Tracing the domesticatin of a biofilm-forming bacterium. J Bacteriol 193: 2027–2034 10.1128/JB.01542-10 21278284 PMC3133032

[27] Warner FD, Kitos GA, Romano MP, Hemphill HE (1977) Characterization of SPβ: a temperate bacteriophage from Bacillus subtilis 168M. Can J Microbiol 23: 45–51 10.1139/m77-006

[28] Vagner V, Dervyn E, Ehrlich SD (1998) A vector for systematic gene inactivation in Bacillus subtilis. Microbiology 144: 3097–3104 10.1099/00221287-144-11-3097 9846745

[29] Sacco M, Ricca E, Losick R, Cutting S (1995) An additional GerE-controlled gene encoding an abundant spore coat protein from Bacillus subtilis. J Bacteriol 177: 372–377. 7814326 10.1128/jb.177.2.372-377.1995 PMC176600

[30] Sierro N, Makita Y, de Hoon M, Nakai K (2008) DBTBS: a database of transcriptional regulation in Bacillus subtilis containing upstream intergenic conservation information. Nucleic Acids Res 36: D93–D96 10.1093/nar/gkm910 17962296 PMC2247474

[31] Aucken HM, Wilkinson SG, Pitt TL (1997) Identification of capsular antigens in Serratia marcescens. J Clin Microbiol 35: 59–63. 8968881 10.1128/jcm.35.1.59-63.1997 PMC229512

[32] Hammerschmidt S, Wolff S, Hocke A, Rosseau S, Müller E, et al (2005) Illustration of pneumococcal polysaccharide capsule during adherence and invasion of epithelial cells. Infect Immun 73: 4653–4667 10.1128/IAI.73.8.4653-4667.2005 16040978 PMC1201225

[33] Wunschel D, Fox KF, Black GE, Fox A (1994) Discrimination among the B. cereus group, in comparison to B. subtilis, by structural carbohydrate profiles and ribosomal RNA spacer region PCR. Syst Appl Microbiol 17: 625–635 10.1016/S0723-2020(11)80085-8

[34] Plata G, Fuhrer T, Hsiao TL, Sauer U, Vitkup D (2012) Global probabilistic annotation of metabolic networks enables enzyme discovery. Nat Chem Biol 8: 848–854 10.1038/nchembio.1063 22960854 PMC3696893

[35] Takemaru K, Mizuno M, Sato T, Takeuchi M, Kobayashi Y (1995) Complete nucleotide sequence of a skin element excised by DNA rearrangement during sporulation in Bacillus subtilis. Microbiology 141: 323–327 10.1099/13500872-141-2-323 7704261

[36] Kunkel B, Losick R, Stragier P (1990) The Bacillus subtilis gene for the development transcription factor σ^K^ is generated by excision of a dispensable DNA element containing a sporulation recombinase gene. Genes Dev 4: 525–535 10.1101/gad.4.4.525 2163341

[37] Lu S, Halberg R, Kross L (1990) Processing of the mother-cell σ factor, σ^K^, may depend on events occurring in the forespore during Bacillus subtilis development. Proc Natl Acad Sci USA 87: 9722–9726. 2124700 10.1073/pnas.87.24.9722 PMC55245

[38] Paik SH, Chakicherla A, Hansen JN (1998) Identification and characterization of the structural and transporter genes for, and the chemical and biological properties of, sublancin 168, a novel lantibiotic produced by Bacillus subtilis 168. J Biol Chem 273: 23134–23142 10.1074/jbc.273.36.23134 9722542

[39] Matsuoka S, Arai T, Murayama R, Kawamura F, Asai K, et al (2004) Identification of the nonA and nonB loci of Bacillus subtilis Marburg permitting the growth of SP10 phage. Genes Genet Syst 79: 311–317 10.1266/ggs.79.311 15728999

[40] Yee LM, Matsuoka S, Yano K, Sadaie Y, Asai K (2011) Inhibitory effect of prophage SPβ fragments on phage SP10 ribonucleotide reductase function and its multiplication in Bacillus subtilis. Genes Genet Syst 86: 7–18 10.1266/ggs.86.7 21498918

[41] Yamamoto T, Obana N, Yee LM, Asai K, Nomura N, et al (2014) SP10 infectivity is aborted after bacteriophage SP10 infection induces nonA transcription on prophage SPβ region of Bacillus subtilis genome. J Bacteriol 196: 693–706 10.1128/JB.01240-13 24272782 PMC3911148

[42] Rabinovich L, Sigal N, Borovok I, Nir-Paz R, Herskovits AA (2012) Prophage excision activates Listeria competence genes that promote phagosomal escape and virulence. Cell 150: 792–802 10.1016/j.cell.2012.06.036 22901809

[43] Lewis JA, Hatfull GF (2001) Control of directionality in integarse-mediated recombination: examination of recombination directionality factors (RDFs) including Xis and Cox proteins. Nucleic Acids Res 29: 2205–2216 10.1093/nar/29.11.2205 11376138 PMC55702

[44] Matz LL, Beaman TC, Gerhard P (1970) Chemical composition of exosporium from spores of Bacillus cereus. J Bacteriol 10: 196–201. 10.1128/jb.101.1.196-201.1970 PMC250470 4983648

[45] Fox A, Stewart GC, Waller LN, Fox KF, Harley WM, et al (2003) Carbohydrates and glycoproteins of Bacillus anthracis and related Bacilli: targets for biodetection. J Microbiol Methods 54: 143–152 10.1016/S0167-7012(03)00095-2 12782370

[46] Zhang J, Fitz-James PC, Aronson AI (1993) Cloning and characterization of a cluster of genes encoding polypeptides present in the insoluble fraction of the spore coat of Bacillus subtilis. J Bacteriol 175: 3757–3766. 8509331 10.1128/jb.175.12.3757-3766.1993 PMC204792

[47] Harwood CR, Cutting SS. (1990) Molecular Biological Methods for Bacillus. Chichester: John Wiley & Sons Ltd.

[48] Murakami T, Haga K, Takeuchi M, Sato T (2002) Analysis of the Bacillus subtilis spoIIIJ Gene and Its Paralogue Gene, yqjG. J Bacteriol 184: 1998–2004 10.1128/JB.184.7.1998-2004.2002 11889108 PMC134917

[49] Mason JM, Hackett RH, Setlow P (1988) Regulation of expression of genes coding for small, acid-soluble proteins of Bacillus subtilis spores: studies using lacZ gene fusions. J Bacteriol 170: 239–244. 3121585 10.1128/jb.170.1.239-244.1988 PMC210633

[50] Miller JM. (1972) Experiments in molecular genetics. New York: Cold Spring Harbor Laboratory Press. pp.352–355.

[51] Abe K, Obana N, Nakamura K (2010) Effects of depletion of RNA-binding protein Tex on the expression of toxin genes in Clostridium perfringens. Biosci Biotechnol Biochem 74: 1564–1571 10.1271/bbb.100135 20699586

[52] Keggins KM, Lovett PS, Duvall EJ (1978) Molecular cloning of genetically active fragments of Bacillus DNA in Bacillus subtilis and properties of the vector plasmid pUB110. Proc Natl Acad Sci U S A 75: 1423–1427. 418411 10.1073/pnas.75.3.1423 PMC411484

[53] Hosoya S, Asai K, Ogasawara N, Takeuchi M, Sato T (2002) Mutation in yaaT leads to significant inhibition of phosphorelay during sporulation in Bacillus subtilis. J Bacteriol 184: 5545–5553 10.1128/JB.184.20.5545-5553.2002 12270811 PMC139598

[54] Carrera M, Zandomeni RO, Fitzgibbon J, Sagripanti JL (2007) Difference between the spore sizes of Bacillus anthracis and other Bacillus species. J App Microbiol 102: 303–312 10.1111/j.1365-2672.2006.03111.x 17241334

