# Supplementary figures and images for: Developmentally-Regulated Excision of the SPβ Prophage Reconstitutes a Gene Required for Spore Envelope Maturation in Bacillus subtilis
Source: PLoS Genet. 2014 Oct 9;10(10):e1004636. doi: 10.1371/journal.pgen.1004636 (PMC4191935; doi:10.1371/journal.pgen.1004636)

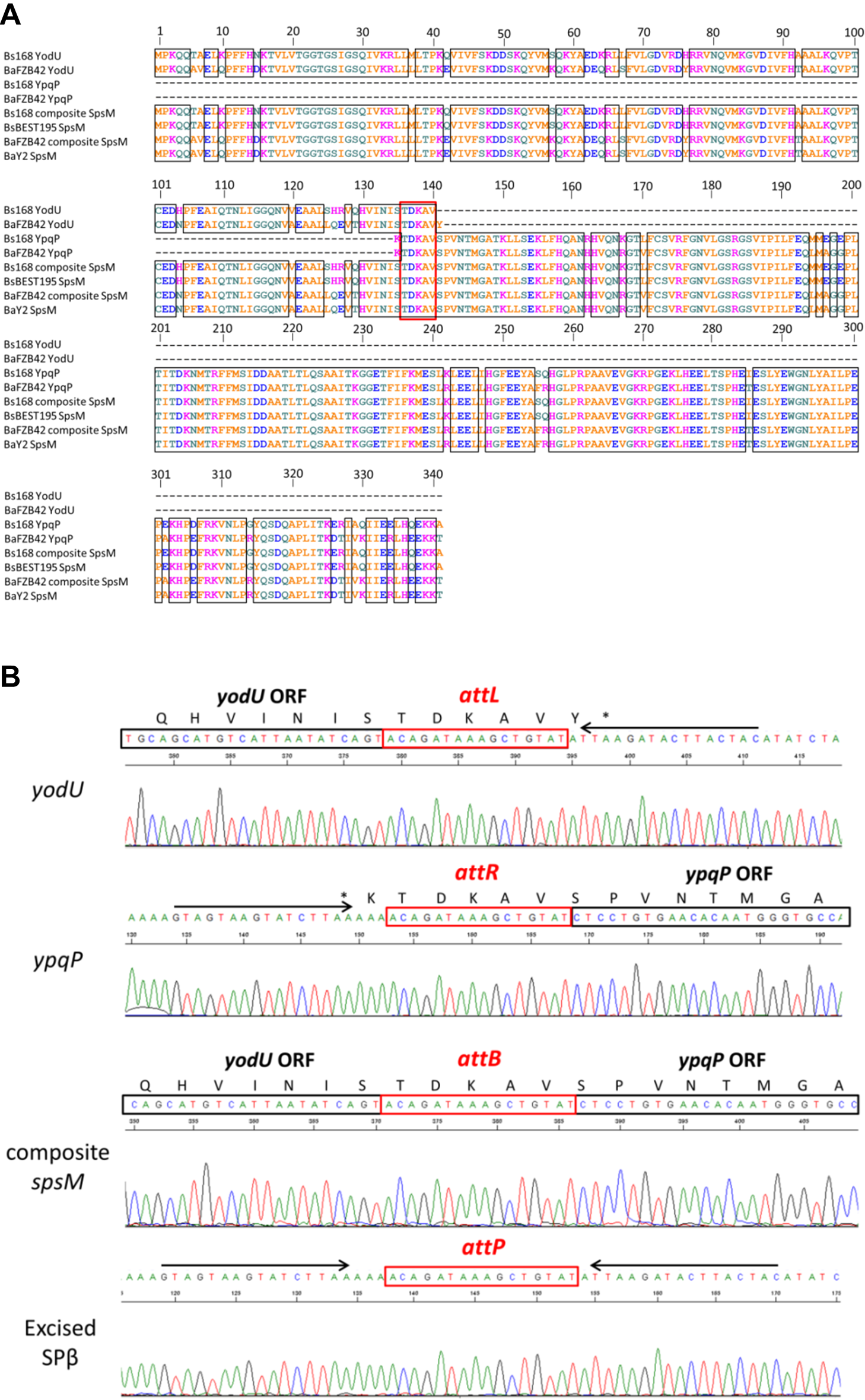

Supplement: Figure S1 — Amino-acid sequence alignment of SpsM proteins. (A) Multiple alignments of SpsM proteins. Amino-acids sequences of YodU, YpqP, and SpsM proteins are shown. The over-lapped amino-acids sequences between YodU and YpqP of B. subtilis 168 are boxed in red. Bs168, B. subtilis 168 (YodU, NCBI locus tagBSU19810; YpqP, BSU21670); BsBEST195, B. subtilis BEST195 (SpsM, BSNT03232); BaFZB42, B. amyloliquefaciens FZB42 (YodU, RBAM019650; YpqP, RBAM019840); BaY2, B. amyloliquefaciens Y2 (SpsM, MUS2345). (B) Nucleotides sequences of the joint site of the composite spsM gene. Nucleotide sequences of the attachment sites of SPβ and the joint site of spsM of B. subtilis 168 before and after the DNA rearrangement during sporulation were determined. The 16-bp inverted repeat sequences were indicated by arrows. The sequences boxed in red are core sequences. (TIF) [file pgen.1004636.s001.tif]

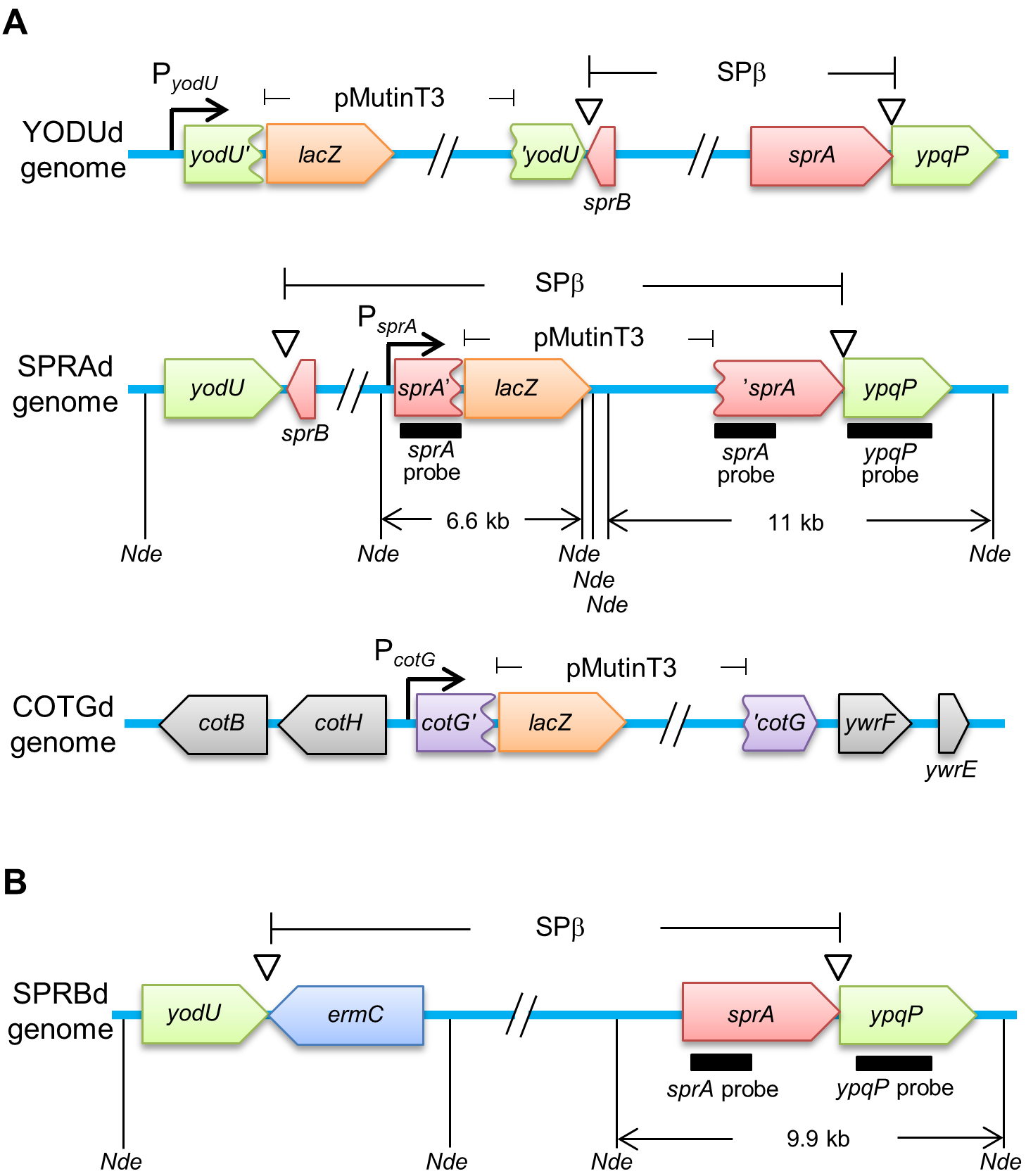

Supplement: Figure S2 — Strain constructs. Schematic drawing showing the yodU, sprA, and cotG gene disruptions by pMutinT3 [upper line: yodU (YODUd); middle line: sprA (SPRAd); bottom line: cotG (COTGd)] (A), and the sprB gene deletion by the ermC cassette (SPRBd) (B). Thick lines indicate the sprA and ypqP probes for Southern blotting. Nde denotes NdeI restriction sites. (TIF) [file pgen.1004636.s002.tif]

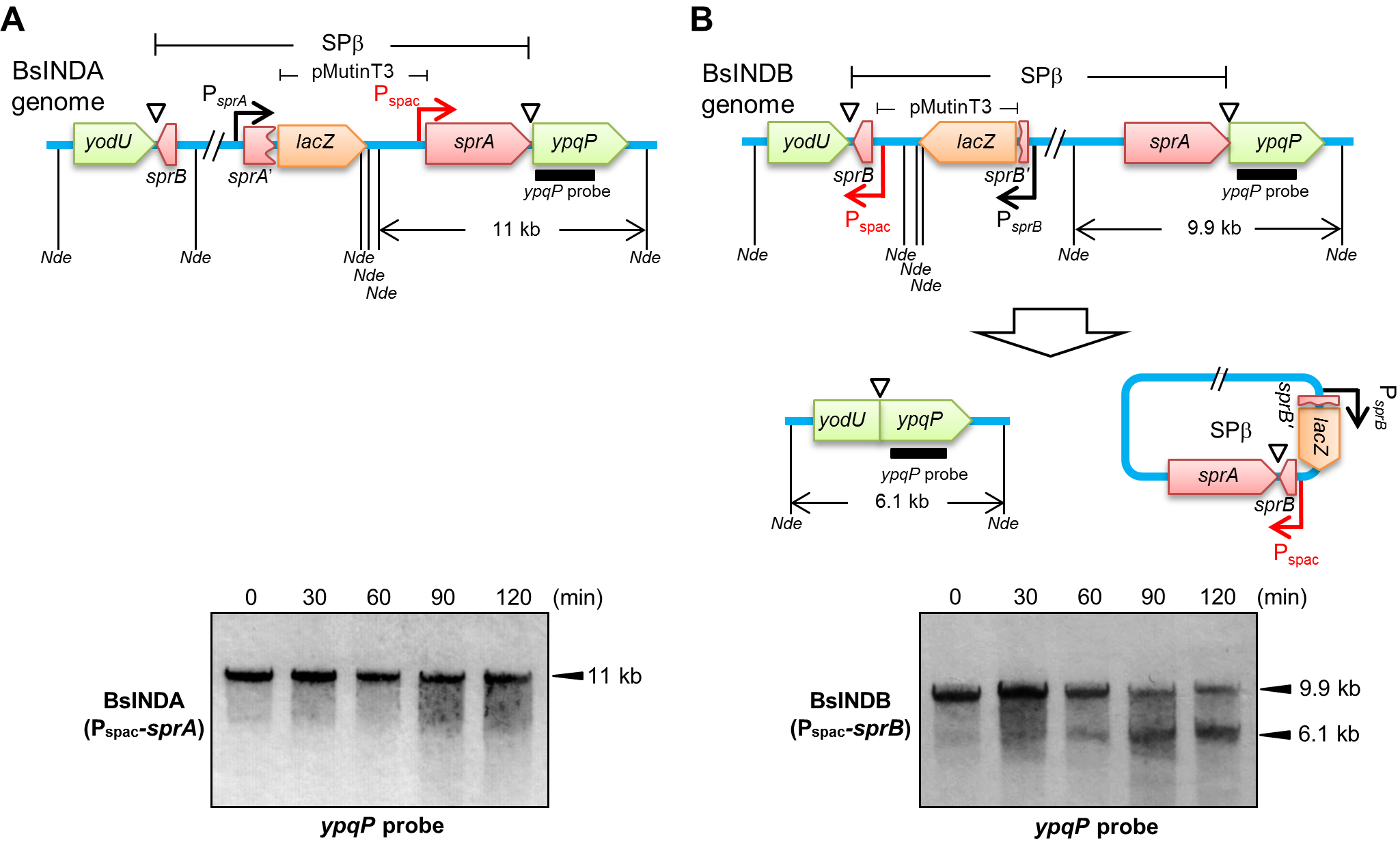

Supplement: Figure S3 — Effects of overexpression of sprA and sprB on the SPβ excision. A schematic above shows the construct of BsINDA (A) and BsINDB (B). The B. subtilis strains carrying the constructs of Pspac–sprA (BsINDA) and Pspac–sprB (BsINDB) were cultured at 37°C in LB medium. IPTG (0.2 mM) was added to the medium when the cells reached mid-log phase of cell growth (OD600 = 0.5). DNA was extracted from the cells at various time points after addition of IPTG and digested with NdeI and subjected to Southern blot using the ypqP probe. (TIF) [file pgen.1004636.s003.tif]

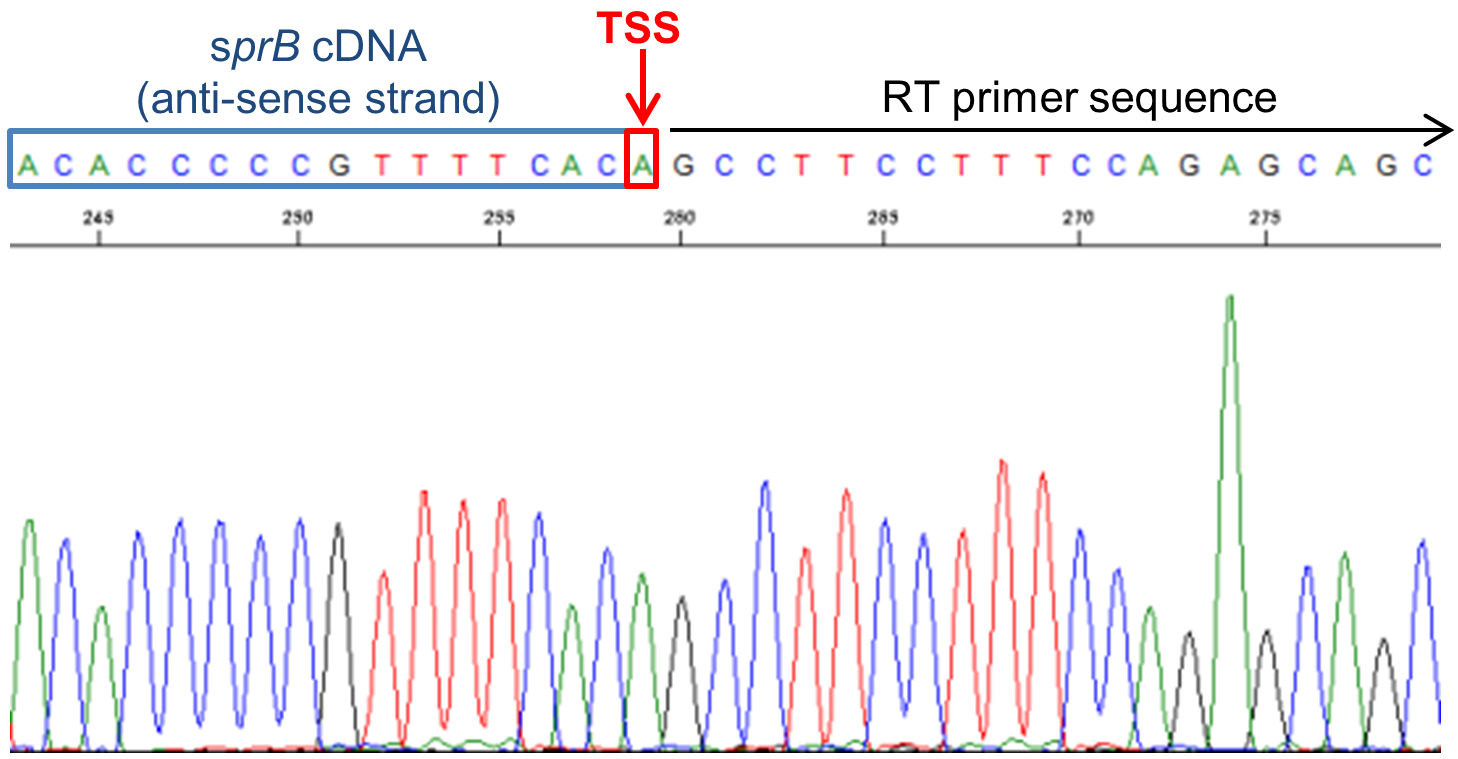

Supplement: Figure S4 — Determination of the transcriptional start site of sprB. 5′ RACE was performed using total RNA from B. subtilis 168 sporulating cells (T4) and the sprB-specific primers. The sequence of the 5′ end of the sprB cDNA is shown. Nucleotides boxed in blue indicate the protein-coding region. The transcriptional start site (TSS) is shown as the nucleotide boxed in red. The black arrow denotes the primer sequence used for the reverse transcription reaction. The predicted sprB promoter region is shown in Figure 5B. (TIF) [file pgen.1004636.s004.tif]

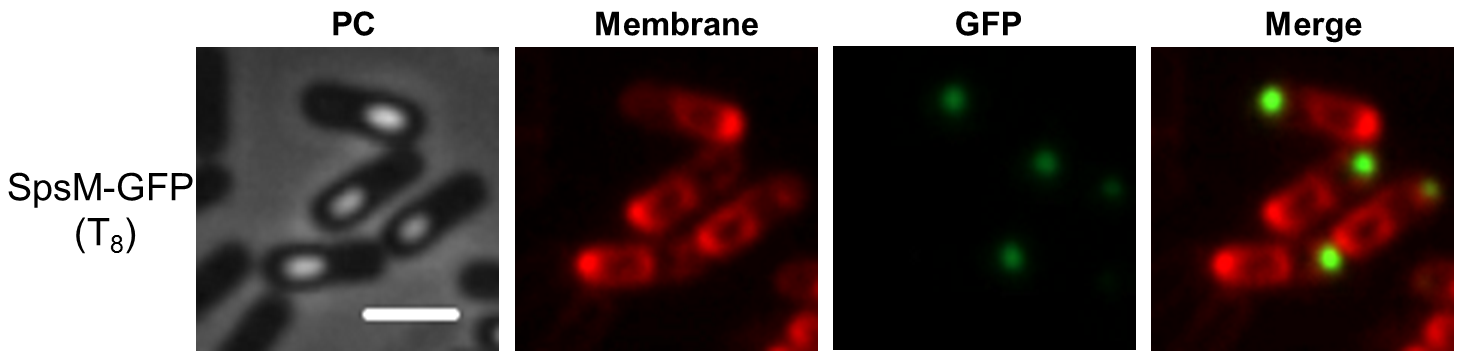

Supplement: Figure S5 — Compartmentalization of SpsM–GFP expression. The B. subtilis strain carrying ypqP–gfp (BsSPSMG) was induced to sporulate at 37°C in liquid DSM containing FM4-64 (0.25 µg/ml). The sporulating cells at T8 were harvested and observed by phase-contrast microscopy. PC, phase-contrast; Membrane, cell membranes stained with FM4-64; GFP, SpsM–GFP; Merge, merged image of Membrane and GFP. Scale bar, 2 µm. (TIF) [file pgen.1004636.s005.tif]

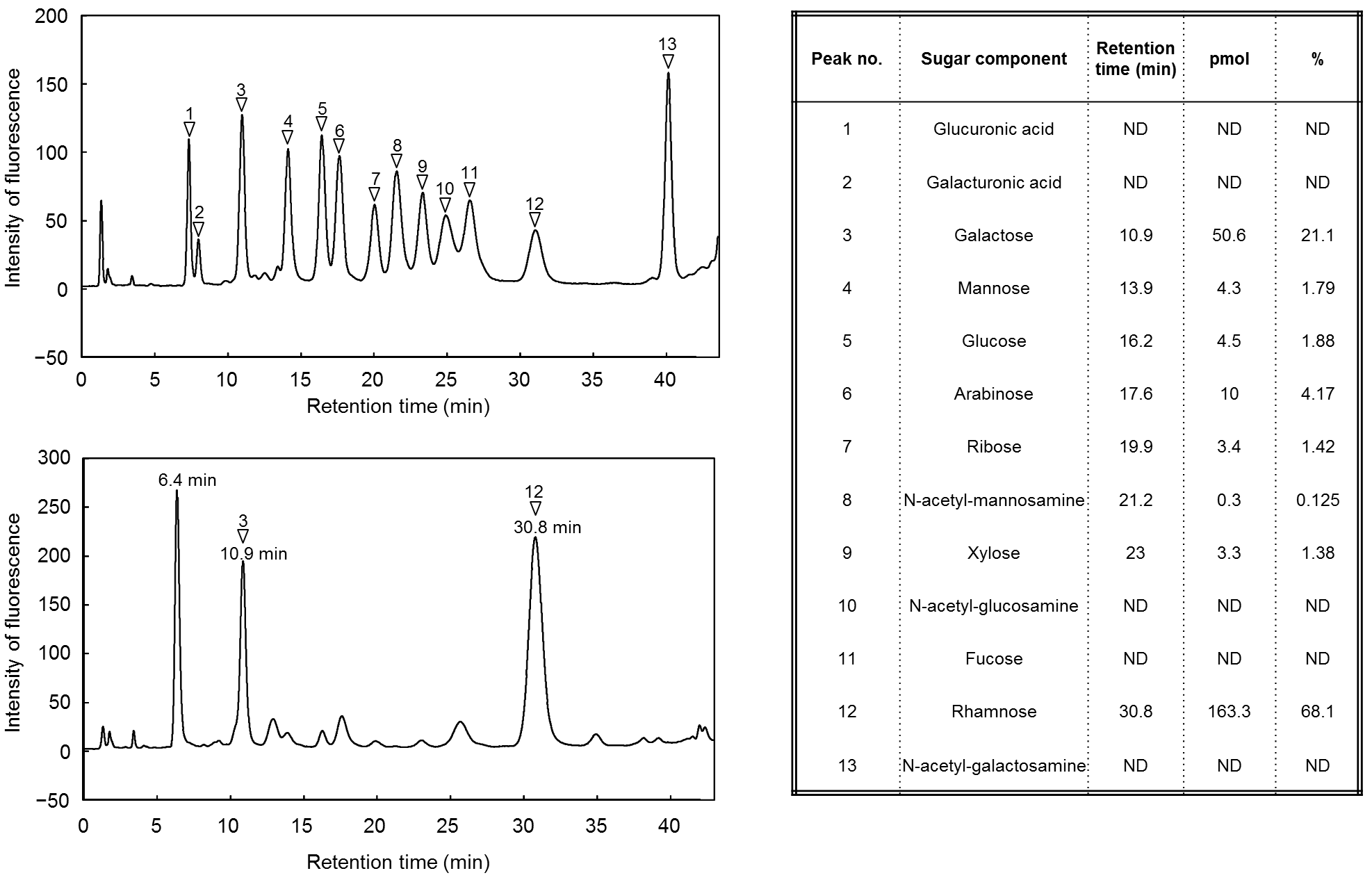

Supplement: Figure S6 — HPLC analysis of the monosaccharide composition of the spore surface polysaccharides. The spore surface polysaccharides from B. subtilis strain 168 spores were hydrolyzed, ABEE-labeled, and loaded onto HPLC. The upper and lower panels show the elution profiles of the standard sugars and the samples, respectively. The x-axis and y-axis indicate the retention time (min) and fluorescence intensity (Ex 305nm, Em 360nm), respectively. The peaks of the standard sugars are indicated by triangles: 1, glucuronic acid; 2, galacturonic acid; 3, galactose; 4, mannose; 5, glucose; 6, arabinose; 7, ribose; 8, N-acetyl-mannosamine; 9, xylose; 10, N-acetyl-glucosamine; 11, fucose; 12, rhamnose; and 13, N-acetyl-galactosamine. We used 29.6 pmol of the standard sugars in the HPLC analysis, except for glucuronic acid and galacturonic acid, i.e., 148.1 pmol of glucuronic acid and galacturonic acid were used for HPLC. (TIF) [file pgen.1004636.s006.tif]
