# Supplementary material for: Developmentally-Regulated Excision of the SPβ Prophage Reconstitutes a Gene Required for Spore Envelope Maturation in Bacillus subtilis
Source: PLoS Genet. 2014 Oct 9;10(10):e1004636. doi: 10.1371/journal.pgen.1004636 (PMC4191935; doi:10.1371/journal.pgen.1004636)
Supplement: Table S1 — Genes in B. amyloliquefaciens SPβ-like elements. (DOCX) [file pgen.1004636.s007.docx]

**Table S1. Genes in *B. amyloliquefaciens* SPβ-like elements.**

| **Gene ID^a^** | **Position** | **Deduced product size (aa)** | **Similar protein^b^** | **Identity (%)** |
| --- | --- | --- | --- | --- |
| *B. amyloliquefaciens* subsp. plantarum NAU-B3 | |  |  |  |
| BAPNAU_1778 | 1846026..1846202 | 58 | SprB | 93.1 |
| BAPNAU_1777 | 1845407..1845664 | 85 | YobA | 47 |
| BAPNAU_1776 | 1844934..1845044 | 36 | YorC | 51.4 |
| BAPNAU_1775 | 1844817..1844933 | 38 | N.S. | - |
| BAPNAU_1774 | 1843447..1843899 | 150 | YoqH | 76.7 |
| BAPNAU_1773 | complement (1841964..1843079) | 371 | RapK | 93.3 |
| BAPNAU_1772 | complement (1841651..1841797) | 48 | N.S. | - |
| BAPNAU_1771 | complement (1840285..1841301) | 338 | YeeG | 59.6 |
| BAPNAU_1770 | 1839865..1840224 | 119 | YolB | 61.4 |
| BAPNAU_1769 | 1839284..1839484 | 66 | YobK | 59.1 |
| BAPNAU_1768 | 1839033..1839257 | 74 | YopB | 77.9 |
| BAPNAU_1767 | 1838502..1838939 | 145 | YoaW | 37.9 |
| BAPNAU_1766 | complement (1837103..1838197) | 364 | OatA | 24.4 |
| BAPNAU_1765 | 1835539..1837044 | 501 | GgaB | 20.7 |
| BAPNAU_1764 | 1835147..1835434 | 95 | Ffh | 27 |
| BAPNAU_1763 | complement (1834178..1834456) | 92 | N.S. | - |
| BAPNAU_1762 | complement (1832900..1834036) | 378 | RapA | 49.1 |
| BAPNAU_1761 | 1832281..1832694 | 137 | UvrX | 88.9 |
| BAPNAU_1760 | 1830591..1832003 | 470 | YrkH | 24.7 |
| BAPNAU_1759 | 1829233..1830432 | 399 | YumB | 22.4 |
| BAPNAU_1758 | 1828412..1829056 | 214 | LiaR | 41.3 |
| BAPNAU_1757 | 1827346..1828419 | 357 | degS | 33.9 |
| BAPNAU_1756 | 1826173..1826898 | 241 | YrkJ | 55.1 |
| BAPNAU_1755 | 1825151..1825516 | 121 | YolB | 67.6 |
| BAPNAU_1754 | 1824040..1824498 | 152 | HisJ | 23.6 |
| BAPNAU_1753 | 1823486..1823947 | 153 | YobK | 61.6 |
| BAPNAU_1752 | 1821707..1823470 | 587 | YobL | 56.5 |
| BAPNAU_1751 | 1821134..1821670 | 178 | YnaB | 78.6 |
| BAPNAU_1750 | complement (1820007..1820972) | 321 | YokF | 59.4 |
| BAPNAU_1749 | 1819551..1819871 | 106 | N.S. | - |
| **Table S1. (*cont.*)** | | | | |
| **Gene ID^a^** | **Position** | **Deduced product size (aa)** | **Similar protein^b^** | **Identity (%)** |
| BAPNAU_1748 | 1818895..1819527 | 210 | YttA | 24.2 |
| BAPNAU_1747 | 1818412..1818603 | 63 | N.S. | - |
| BAPNAU_1746 | complement (1816498..1818165) | 555 | SprA | 89.6 |
|  | | | | |
| *B. amyloliquefaciens* CAU B946 | | | | |
| BACAU_1967 | complement (2166832..2167008) | 58 | SprB | 93.1 |
| BACAU_1968 | complement (2167360..2167635) | 91 | YobA | 44.7 |
| BACAU_1969 | 2170238..2170459 | 73 | LigB | 66.7 |
| BACAU_1970 | 2172464..2172838 | 124 | YoqO | 50.4 |
| BACAU_1971 | complement (2173072..2173464) | 130 | YoqH | 80.5 |
| BACAU_1972 | 2173888..2174262 | 124 | RapH | 66.4 |
| BACAU_1973 | 2174216..2175022 | 268 | RapA | 43.6 |
| BACAU_1974 | 2175520..2176287 | 255 | YeeG | 59.4 |
| BACAU_1975 | 2176369..2176539 | 56 | YeeG | 64.3 |
| BACAU_1976 | complement (2176600..2176959) | 119 | YolB | 60.5 |
| BACAU_1977 | complement (2176965..2177432) | 155 | YolA | 57.1 |
| BACAU_1978 | complement (2178008..2178346) | 112 | YozM | 56.9 |
| BACAU_1979 | complement (2178560..2178718) | 52 | N.S. | - |
| BACAU_1980 | complement (2178752..2179012) | 86 | N.S. | - |
| BACAU_1981 | complement (2179108..2179704) | 198 | YokK | 84.1 |
| BACAU_1982 | complement (2179759..2180217) | 152 | YobK | 57.2 |
| BACAU_1983 | complement (2180232..2182031) | 599 | YobL | 72.6 |
| BACAU_1984 | complement (2182068..2182601) | 177 | YnaB | 81.9 |
| BACAU_1985 | 2182765..2183730 | 321 | YokF | 60 |
| BACAU_1986 | complement (2183861..2184184) | 107 | N.S. | - |
| BACAU_1987 | complement (2184296..2184766) | 156 | N.S. | - |
| BACAU_1988 | 2185502..2187169 | 555 | SprA | 89.4 |
|  |  |  |  |  |
| *B. amyloliquefaciens* FZB42 | |  |  |  |
| RBAM_019660 | complement (2078509..2078685) | 58 | SprB | 93.1 |
| RBAM_019670 | complement (2079047..2079400) | 117 | YobA | 44.2 |
| RBAM_019680 | complement (2079403..2079588) | 61 | N.S. | - |
| **Table S1. (*cont.*)** | | | | |
| **Gene ID^a^** | **Position** | **Deduced product size (aa)** | **Similar protein^b^** | **Identity (%)** |
| RBAM_019690 | complement (2079672..2079782) | 36 | YorC | 56.8 |
| RBAM_019700 | 2080208..2080582 | 124 | YoqO | 51.2 |
| RBAM_019710 | complement (2080615..2080701) | 28 | N.S. | - |
| RBAM_019720 | complement (2080816..2081268) | 150 | YoqH | 77.3 |
| RBAM_019730 | 2081538..2082674 | 378 | RapA | 50.1 |
| RBAM_019740 | complement (2082877..2083506) | 209 | UvrX | 92.8 |
| RBAM_019750 | complement (2083569..2084981) | 470 | YrkH | 25.9 |
| RBAM_019760 | complement (2085140..2086339) | 399 | YumB | 22.7 |
| RBAM_019770 | complement (2086586..2087164) | 192 | LiaR | 43.5 |
| RBAM_019780 | complement (2087157..2088230) | 357 | DegS | 33.9 |
| RBAM_019790 | complement (2088677..2089204) | 175 | YrkJ | 52.5 |
| RBAM_019800 | complement (2089972..2090112) | 46 | YolB | 90.7 |
| RBAM_019810 | complement (2090978..2091538) | 186 | YokK | 40.8 |
| RBAM_019820 | complement (2091539..2092228) | 229 | YokI | 41.1 |
| RBAM_019830 | 2092844..2094511 | 555 | SprA | 90 |
|  |  |  |  |  |
| *B. amyloliquefaciens* CC178 | |  |  |  |
| U471_20310 | complement (2075585..2075761) | 58 | SprB | 93.1 |
| U471_20320 | complement (2076123..2076380) | 85 | YobA | 45.8 |
| U471_20330 | complement (2077606..2077782) | 58 | N.S. | - |
| U471_20340 | 2078614..2079750 | 378 | RapA | 50.1 |
| U471_20350 | complement (2079953..2080207) | 84 | UvrX | 89.3 |
| U471_20360 | complement (2080645..2082057) | 470 | YrkH | 25.9 |
| U471_20370 | complement (2082216..2083415) | 399 | YumB | 22.7 |
| U471_20380 | complement (2083662..2084240) | 192 | LiaR | 43.5 |
| U471_20390 | complement (2084233..2085306) | 357 | DegS | 33.9 |
| U471_20400 | complement (2085753..2085887) | 44 | YrkJ | 48.8 |
| U471_20410 | 2087813..2087926 | 37 | N.S. | - |
| U471_20420 | complement (2088054..2088614) | 186 | YokK | 40.8 |
| U471_20430 | complement (2088615..2089304) | 229 | YokI | 41.1 |
| U471_20440 | 2089920..2091587 | 555 | SprA | 90 |
|  |  |  |  |  |
| **Table S1. (*cont.*)** | | | | |
| **Gene ID^a^** | **Position** | **Deduced product size (aa)** | **Similar protein^b^** | **Identity (%)** |
| *B. amyloliquefaciens* subsp. plantarum UCMB5036 | | | | |
| BAM5036_1899 | complement (2074774..2074950) | 58 | SprB | 91.4 |
| BAM5036_1900 | complement (2075313..2075570) | 85 | YobA | 47 |
| BAM5036_1901 | 2076100..2076336 | 78 | N.S. | - |
| BAM5036_1902 | 2076463..2076837 | 124 | YoqO | 52 |
| BAM5036_1903 | complement (2077058..2077510) | 150 | YoqH | 78.7 |
| BAM5036_1904 | 2077881..2078996 | 371 | RapK | 93 |
| BAM5036_1905 | 2079639..2080658 | 339 | YeeG | 59.9 |
| BAM5036_1906 | complement (2081026..2081262) | 78 | YolA | 55 |
| BAM5036_1907 | complement (2081445..2081909) | 154 | YobK | 53.9 |
| BAM5036_1908 | complement (2081906..2083735) | 609 | YobL | 73.5 |
| BAM5036_1909 | complement (2083772..2084305) | 177 | YnaB | 78.5 |
| BAM5036_1910 | 2084471..2085436 | 321 | YokF | 60.3 |
| BAM5036_1911 | 2085652..2087319 | 555 | SprA | 90.2 |
|  | |  |  |  |
| *B. amyloliquefaciens* subsp. plantarum AS43.3 | |  |  |  |
| B938_10190 | complement (2140838..2141014) | 58 | SprB | 93.1 |
| B938_10195 | complement (2141377..2141730) | 117 | YobA | 43 |
| B938_10200 | 2142187..2142423 | 78 | N.S. | - |
| B938_10205 | 2142549..2142923 | 124 | YoqO | 52 |
| B938_10210 | 2143966..2145102 | 378 | RapA | 48.5 |
| B938_10215 | 2145092..2145274 | 60 | N.S. | - |
| B938_10220 | complement (2145667..2145921) | 84 | YcdD | 33.8 |
| B938_10225 | complement (2146126..2146551) | 141 | N.S. | - |
| B938_10230 | complement (2146681..2147139) | 152 | YobK | 55.3 |
| B938_10235 | complement (2147142..2148947) | 601 | YobL | 72.4 |
| B938_10240 | complement (2148984..2149517) | 177 | YnaB | 78.5 |
| B938_10245 | 2149683..2150648 | 321 | YokF | 60 |
| B938_10250 | 2150865..2152532 | 555 | SprA | 91 |
|  |  |  |  |  |
| *B. amyloliquefaciens* subsp. plantarum UCMB5033 | |  |  |  |
| RBAU_1936 | complement (2105451..2105627) | 58 | SprB | 91.4 |
| **Table S1. (*cont.*)** | | | | |
| **Gene ID^a^** | **Position** | **Deduced product size (aa)** | **Similar protein^b^** | **Identity (%)** |
| RBAU_1937 | complement (2105995..2106354) | 119 | YobA | 41.6 |
| RBAU_1938 | 2106798..2107034 | 78 | N.S. | - |
| RBAU_1939 | 2107160..2107534 | 124 | YoqO | 51.2 |
| RBAU_1940 | complement (2107768..2108220) | 150 | YoqH | 76.7 |
| RBAU_1941 | 2108599..2109714 | 371 | RapK | 92.7 |
| RBAU_1942 | complement (2109869..2110219) | 116 | N.S. | - |
| RBAU_1943 | complement (2110315..2110911) | 198 | YokK | 82.5 |
| RBAU_1944 | complement (2110913..2112664) | 583 | YokI | 61 |
| RBAU_1945 | complement (2112701..2113234) | 177 | YnaB | 79.2 |
| RBAU_1946 | 2113400..2114365 | 321 | YokF | 60.3 |
| RBAU_1947 | 2114582..2116249 | 555 | SprA | 90.8 |
|  |  |  |  |  |
| *B. amyloliquefaciens* TA208 | |  |  |  |
| BAMTA208_07180 | 1349780..1350061 | 93 | SprB | 43.4 |
| BAMTA208_07175 | complement (1349346..1349690) | 114 | YjdB | 33 |
| BAMTA208_07170 | complement (1348013..1349167) | 384 | Fni | 20.9 |
| BAMTA208_07165 | complement (1347771..1347992) | 73 | N.S. | - |
| BAMTA208_07160 | complement (1347270..1347698) | 142 | YmzD | 32.8 |
| BAMTA208_07155 | complement (1345603..1347228) | 541 | SprA | 69.7 |
|  |  |  |  |  |
| *B. amyloliquefaciens* DSM 7 | |  |  |  |
| BAMF_2064 | complement (2157811..2158002) | 63 | SprB | 43.4 |
| BAMF_2065 | 2158182..2158526 | 114 | YjdB | 33 |
| BAMF_2066 | 2158705..2159859 | 384 | Fni | 20.9 |
| BAMF_2067 | 2159877..2160101 | 74 | N.S. | - |
| BAMF_2068 | 2160174..2160602 | 142 | YmzD | 32.8 |
| BAMF_2069 | 2160644..2162269 | 541 | SprA | 69.7 |
|  | |  |  |  |
| *B. amyloliquefaciens* LL3 | |  |  |  |
| LL3_02168 | complement (2102538..2102708) | 56 | SprB | 43.4 |
| LL3_02169 | 2102909..2103253 | 114 | YjdB | 33 |
| LL3_02170 | 2103432..2104586 | 384 | Fni | 20.9 |
| **Table S1. (*cont.*)** | | | | |
| **Gene ID^a^** | **Position** | **Deduced product size (aa)** | **Similar protein^b^** | **Identity (%)** |
| LL3_02171 | 2104607..2104828 | 73 | N.S. | - |
| LL3_02172 | 2104901..2105329 | 142 | YmzD | 32.8 |
| LL3_02173 | 2105371..2106996 | 541 | SprA | 69.7 |
|  |  |  |  |  |
| *B. amyloliquefaciens* XH7 | |  |  |  |
| BAXH7_01460 | 1351468..1351638 | 56 | SprB | 43.4 |
| BAXH7_01459 | complement (1350923..1351267) | 114 | YjdB | 33 |
| BAXH7_01458 | complement (1349590..1350744) | 384 | Fni | 20.5 |
| BAXH7_01457 | complement (1349348..1349569) | 73 | N.S. | - |
| BAXH7_01456 | complement (1348847..1349275) | 142 | YmzD | 32.8 |
| BAXH7_01455 | complement (1347180..1348805) | 541 | SprA | 69.7 |

^a^Locus tags of genes located in the *B. amyloliquefaciens* SPβ-like elements are shown. All of the SPβ-like elements from *B. amyloliquefaciens* strains deposited in the KEGG genome database (http://www.genome.jp/kegg/) were analyzed and listed on 23 December 2013.
