# Supplementary material for: Developmentally-Regulated Excision of the SPβ Prophage Reconstitutes a Gene Required for Spore Envelope Maturation in Bacillus subtilis
Source: PLoS Genet. 2014 Oct 9;10(10):e1004636. doi: 10.1371/journal.pgen.1004636 (PMC4191935; doi:10.1371/journal.pgen.1004636)
Supplement: Table S2 — Sporulation frequencies of B. subtilis strains. (DOCX) [file pgen.1004636.s008.docx]

**Table S2. Sporulation frequencies of *B. subtilis* strains.**

| Strain | Viable cells (ml^−1^)*^a^* | Spores (ml^−1^)*^b^* | Sporulation frequency*^c^* |
| --- | --- | --- | --- |
| 168 (WT) | 4.6 × 10^8^ | 3.7 × 10^8^ | 0.80 |
| SPRAd (*sprA*) | 3.3 × 10^8^ | 1.6 × 10^8^ | 0.48 |
| YODUd (*yodU*) | 2.6 × 10^8^ | 1.3 × 10^8^ | 0.50 |
| SPRAc (*sprA spsM*^+^) | 5.5 × 10^8^ | 4.1 × 10^8^ | 0.75 |
| YODUc (*yodU spsM*^+^) | 5.2 × 10^8^ | 4.7 × 10^8^ | 0.90 |
| SPless | 4.5 × 10^8^ | 3.3 × 10^8^ | 0.73 |

*^a^B. subtilis* strains were cultured up to T_24_ at 37°C in 5 ml of liquid DSM.

*^b^*Number of spores were determined by counting heat-resistant colonies (80°C for 10 min) on the LB plates.

*^c^*Sporulation frequency is the ratio of the number of spores to that of viable cells for each strain.
