# Supplementary material for: Developmentally-Regulated Excision of the SPβ Prophage Reconstitutes a Gene Required for Spore Envelope Maturation in Bacillus subtilis
Source: PLoS Genet. 2014 Oct 9;10(10):e1004636. doi: 10.1371/journal.pgen.1004636 (PMC4191935; doi:10.1371/journal.pgen.1004636)
Supplement: Table S3 — Primers used in this study. (DOCX) [file pgen.1004636.s009.docx]

**Table S3. Primers used in this study.**

| Name | Sequence (5′ to 3′)*^a^* | Location*^b^* and restriction site*^c^* |
| --- | --- | --- |
| P01 | AGCA*A*A*G*CT*T*AAACCATTTTTCCATAACAA | *yodU* sense strand +47, *Hin*dIII |
| P02 | CCTT*GG*ATC*C*CCTGATTCACCCTTCTGTGA | *yodU* anti-sense strand +225, *Bam*HI |
| P03 | GTTAA*AG*CTTACAACATCACAAATATTTTA | *sprA* sense strand +48, *Hin*dIII |
| P04 | GTTT*G*GAT*C*CGTATTTAGATAAAGGCTCAC | *sprA* anti-sense strand +971, *Bam*HI |
| P05 | TTCC*A*A*GCT*TGACATCGAAGAAGCGGTGAAATCCG | *cotG* sense strand +46, *Hin*dIII |
| P06 | TTGTGG*A*T*C*CGAGATTTTTTGTGAGAACAGAATGA | *cotG* anti-sense strand +193, *Bam*HI |
| P07 | TTT*A*A*GC*TTAAACACAAATAAAGAGGTGCT | *sprA* sense strand −7, *Hin*dIII |
| P08 | TAATA*AG*C*T*TGTGAAAACGGGGGTGTAGCC | *sprB* sense strand −1, *Hin*dIII |
| P09 | TGCT*GG*ATCCTGAGTTATATATCGAATACT | *sprB* anti-sense strand +70, *Bam*HI |
| P10 | ATGCCTAAACAGCAAACAGC | *yodU* sense strand +20 |
| P11 | TGATGCAATTCTTCAATAAT | *ypqP* anti-sense strand +571 |
| P12 | TAATGAA*T*T*C*AATATTGCACATATGGGAAA | *sprB* sense strand −1107 |
| P13 | *CCATGCGTTTGGGCC*GGCTACACCCCCGTT | *sprB* anti-sense strand −15 |
| P14 | *CCAAGGAGATGGCCG*GCAGAGTAAGCTTCT | *sprB* sense strand +183 |
| P15 | ATTCATCTGGCGCTTTCTTG | *sprB* anti-sense strand +2227 |
| P16 | *GGCCCAAACGCATGG*TAAACGTATATAGAT | pUCE191 *ermC* sense strand −370 |
| P17 | *CGGCCATCTCCTTGG*TCGCGCGTTTCGGTG | pUCE191 *ermC* anti-sense strand +973 |
| P18 | *CCATGCGTTTGGGCC*AAGCAACACAATACC | *sprB* sense strand −317 |
| P19 | TATTGAGCTTGCCAAACTCATAAGAATGAA | *sprB* anti-sense strand +1272 |
| P20 | *CCAAGGAGATGGCCG*TGTTTTCGTATTCAA | *sprA* sense strand −70 |
| P21 | TTTTG*A*A*TT*CTTCATTTGGTACCCTCCTCT | *yodU* sense strand −355, *Eco*RI |
| P22 | CTGCA*GA*TCTTTAATAAATAAACTGTTAAA | *ypqP* anti-sense strand +641, *Bgl*II |
| P23 | TGAAAAGCT*T*TTTCATCAAGCAAACCGCCA | *ypqP* sense strand +68, *Hin*dIII |
| P24 | ACCTGGA*TC*CTTGCCCACTTCTACTGCAGC | *ypqP* anti-sense strand +367, *Bam*HI |
| P25 | *TCCACCTCCGCCTCC*CTCTGCCTTCCTTTCCA | *sprB* anti-sense strand +158 |
| P26 | TTGGAATTAAAAAACATCGT | *sprA_Bam_* sense strand +20 |
| P27 | GTTAAAGATGAATTGAACGA | *sprA_Bam_* anti-sense strand +581 |
| P28 | ATGGGAGCTACGAAGCTGTT | *ypqP_Bam_* sense strand +20 |
| P29 | AGATTGACCTTCCGAAATTC | *ypqP_Bam_* anti-sense strand +481 |
| P30 | 5′ Phos–GCCTTCCTTTCCAGAGCAGC | *sprB* anti-sense strand +151 |
| P31 | TTGGAAGTAGGAGACAAAATACATAACACC | *yosX* sense strand +30 |
| P32 | TCAGATATCATTTAATTGTTGTTTCAGCTC | *yosX* anti-sense strand +325 |
| P33 | GATTA*A*G*C*TTCAGATCTCCATATAAATCATTGGA | *yotB* sense strand +43, *Hin*dIII |
| P34 | CTGAATTGTCACCTATCCAACTAA | *yotD* anti-sense strand +47 |
| P35 | *GGAGGCGGAGGTGGA*AGTAAAGGAGAAGAA | pMF20 *gfp* sense strand +18 |
| P36 | *ACCGCCTTTGAGTGA*AATAGTACATAATG | pMF20 *gfp* anti-sense strand +818 |
| P37 | *TTCACTGGCCGTCGT*GAACGAGACTTTGCA | pUB110 *bleO* sense strand +344 |
| P38 | *TCACTCAAAGGCGGT*GATAGACTGTAACAT | pUB110 *bleO* anti-sense strand +1 |
| P39 | AAACAATAAAGGTAGTCCAG | *yodU* anti-sense strand +581 |
| P40 | AAAGAACGAGCTATTAAGGA | *ypqP* sense strand −171 |
| P41 | GTAATGACTTAGATGAGTTTTATAA | *sprB* sense strand +125 |
| P42 | TTGCTTCATCCTGAGTTATATATCG | *sprB* anti-sense strand +76 |
| P43 | CCATTCAGGCTGCGCAACTG | pMD20 *lacZα* anti-sense strand +256 |
| P44 | CTGTGAA*TT*CAATGGGTGCCACCAAGCTGT | *ypqP* sense strand +34, *Eco*RI |
| P45 | *TCCACCTCCGCCTCC*TGCCTTTTTCTCTTG | *ypqP* anti-sense strand +589 |

*^a^*Additional and mutated sequences that do not correspond to the sequences of the gene were indicated by italic type.

*^b^*The locations indicated the 3′ end positions of the primers relative to the first nucleotides of the coding sequences.

*^c^*The recognition sites for restriction enzymes were indicated by underlines.
