# Supplementary material for: Developmentally-Regulated Excision of the SPβ Prophage Reconstitutes a Gene Required for Spore Envelope Maturation in Bacillus subtilis
Source: PLoS Genet. 2014 Oct 9;10(10):e1004636. doi: 10.1371/journal.pgen.1004636 (PMC4191935; doi:10.1371/journal.pgen.1004636)
Supplement: Table S4 — Strains and plasmids used in this study. (DOCX) [file pgen.1004636.s010.docx]

**Table S4. Strains and plasmids used in this study.**

| Strain or Plasmid | Genotype and/or Relevant Features | Source or Reference |
| --- | --- | --- |
| Strains |  |  |
| *Bacillus subtilis* |  |  |
| 168 | *trpC2* | [55] |
| YODUd | *trpC2*, *yodU*::pMutinT3(P*_yodU_–lacZ*) | This study |
| SPRAd | *trpC2*, *sprA*::pMutinT3(P*_sprA_–lacZ*) | This study |
| COTGd | *trpC2*, *cotG*::pMutinT3(P*_cotG_–lacZ*) | This study |
| BsINDA | *trpC2*, *sprA*::pMutinT3(P*_sprA_–lacZ*, P_spac_*–sprA*) | This study |
| BsINDB | *trpC2*, *sprB*::pMutinT3(P*_sprB_–lacZ*, P_spac_*–sprB*) | This study |
| SPless | SPβ-cured strain derived from BsINDB; *trpC2*, *attB*_SPβ_ | This study |
| SPRBd | *trpC2*, Δ*sprB*::*ermC* | This study |
| SPmini | 168 carrying the minimized SPβ | This study |
| YODUc | *trpC2*, *yodU*::pMutinT3(P*_yodU_–lacZ*), *amyE*::pMFspsM | This study |
| SPRAc | *trpC2*, *sprA*::pMutinT3(P*_sprA_–lacZ*), *amyE*::pMFspsM | This study |
| CU1050 | SPβ sensitive strain; *leuB8*, *thr-5*, *trmS-Lys3*, *metA5*, *attB*_SPβ_ | BGSC*^a^* |
| CU1050 (SPβ) | SPβ-lysogen derived from CU1050 | This study |
| BsSPRBG | 168 carrying pUBsprBgfp | This study |
| BsSPSMG | *trpC2*, *ypqP*:: pUCEypqP-gfp (*ypqP–gfp*) | This study |
|  |  |  |
| *Bacillus amyloliquefaciens* |  |  |
| FZB42 |  | BGSC*^a^* |
|  |  |  |
| *Escherichia coli* |  |  |
| DH5α | F^-^, Φ80d*lacZ*ΔM15, Δ(*lacZYA*–*argF*)U169, *deoR*, *recA1*, *endA1*, *hsdR17(r_k_^−^, m_k_^+^)*, *phoA*, *supE44*, *λ^−^*, *thi-1*, *gyrA96*, *relA1* | Invitrogen |
|  |  |  |
| plasmids |  |  |
| pMutinT3 | P_spac_ (an IPTG inducible promoter), *lacZ*, *lacI^q^*, *bla*, *erm^r^* | [28] |
| pMUT-yodU | pMutinT3 carrying a *yodU* internal segment | This study |
| pMUT-sprA | pMutinT3 carrying a *sprA* internal segment | This study |
| pMUT-cotG | pMutinT3 carrying a *cotG* internal segment | This study |
| pMUT-sprAind | pMutinT3 carrying a *sprA* internal segment | This study |
| pMUT-sprBind | pMutinT3 carrying a *sprB* internal segment | This study |
| pUCE191 | pUC19 carrying *ermC*; *bla*, *lacZ*α, *ermC* | Hosoya, S |
| pMD20 | T-vector derived from pUC19 backbone; *bla*, *lacZ*α | Takara |
| pMF20 | *B. subtilis* integration vector at the *amyE* locus; *cat*, *gfp*, *bla* | [48] |
| pMFspsM | pMF20 carrying composite *spsM* | This study |
| pUCEypqP-gfp | pUCE191 carrying a *ypqP–gfp* translational fusion gene | This study |
| pUB110 | pUB110 *ori*, *repC*, *kan^r^*, *mob*, *bleO* | [52] |
| pUBsprBgfp | pUB110 (Δ*bleO*) carrying *sprB*–*gfp* | This study |

*^a^*BGSC, *Bacillus* Genetic Stock Center, Ohio State University.
