# Supplementary material for: Developmentally-Regulated Excision of the SPβ Prophage Reconstitutes a Gene Required for Spore Envelope Maturation in Bacillus subtilis
Source: PLoS Genet. 2014 Oct 9;10(10):e1004636. doi: 10.1371/journal.pgen.1004636 (PMC4191935; doi:10.1371/journal.pgen.1004636)
Supplement: Text S1 — Supporting materials and methods and references. (DOCX) [file pgen.1004636.s011.docx]

**Supporting Text S1**

**Supporting Materials and Methods**

**DNA sequencing.**

DNA fragments containing the SPβ attachment sites prior and posterior to the DNA rearrangement during sporulation were amplified using primer sets P10/P39 (for amplification of the *attL* site), P40/P11 (for *attR*), P10/P11 (for *attB*), and P40/P39 (for *attP*), respectively. PCR products were gel-purified, fluorescence-labeled using BigDye^®^ Terminator version 3.1 (ABI) with the primers P10 (for *attL* and *attB*) and P40 (for *attR* and *attP*), and sequenced using an ABI PRISM3100^®^ Genetic Analyzer according to the manufactory’s instructions.

**5′ RACE.**

Total RNA was extracted from the *B. subtilis* 168 sporulating cells at T_4_ as described in the Materials and Method section of the main text. The cDNA of the *sprB* mRNA was synthesized by reverse transcription using 5 µg of total RNA, the 5′-phosphorylated *sprB*-specific primer P30, and a 5′-Full RACE Core Set (Takara), according to the manufacturer’s instructions. The 5′ end of the s*prB* cDNA was self-ligated with T4 RNA ligase and amplified by PCR using the primer pair P41/P42. PCR products were cloned into a pMD20 T-vector (Takara) using a Mighty TA-cloning kit (Takara). DNA-sequencing was performed using an ABI PRISM3100^®^ Genetic Analyzer with the P43 primer according to the manufacturer’s instructions.

**Construction of *B. subtilis* strain expressing SpsM–GFP.**

For construction of a SpsM**–**GFP expressing strain, a DNA fragment corresponding to the internal segment of *ypqP* was amplified from *B. subtilis* chromosomal DNA using the P44/P45 primers. An 858-bp DNA fragment of *gfp* was amplified from pMF20 using the primer pair, P35/P36. The resulting PCR products of *ypqP* and *gfp* were combined by OE-PCR with the primer set, P44/P36, digested with *Eco*RI and *Bam*HI, and inserted into the *Eco*RI–*Bam*HI site of pUCE191. The resulting pUCEypqP-gfp was introduced into *B. subtilis* 168-competent cell. Transformants were selected on LB agar plates containing 0.3 μg/ml erythromycin.

**Monosaccharide composition analysis of the spore surface extracts.**

*B. subtilis* wild-type cells were induced to sporulate on three 90-mm DSM-agar plates. The spores were collected from the plates, and the spore surface extract was prepared as described above. The extract was dialyzed overnight against 50 mM phosphate sodium buffer (pH 7.0), precipitated with ethanol, and dried. The pellet was dissolved in 100 μl of DDW and sonicated for 10 min. Subsequently, 50 μl of the sample was added to 50 μl of 8 M trifluoroacetic acid, hydrolyzed at 100°C for 3 h, dried, and dissolved in 100 μl of DDW. The sample was N-acetylated, ABEE-labeled, and analyzed by HPLC, as described previously [56].

**Supporting References**

55. Burkholder PR, Giles NH Jr. (1947) Induced biochemical mutations in Bacillus subtilis. Am J Bot 34: 345–348.

56. Yasuno S, Murata T, Kokubo K, Yamaguchi T, Kamei M. (1997) Two-mode Analysis by High-performance Liquid Chromatography of ρ-Aminobenzoic Ethyl Ester-derivatized Monosaccharides. Biosci Biotechnol Biochem 61: 1944–1946. doi: 10.1271/bbb.61.1944.
